# Effects of sensor geometry, placement, and cycle detection on wearable respiration monitoring with textile printed strain sensors

**DOI:** 10.1007/s44397-026-00054-0

**Published:** 2026-03-27

**Authors:** Manuel Reis Carneiro, João Silva, Mahmoud Tavakoli

**Affiliations:** 1 Department of Health Sciences and Technology, ETH Zürich, 8008 Zürich, Switzerland; 2 Department of Electrical and Computer Engineering, Institute of Systems and Robotics, University of Coimbra, 3030-290 Coimbra, Portugal

**Keywords:** Wearables, E-textiles, Respiration rate, Printed strain sensors, Ag-In-Ga-SIS, Peaks-and-valleys, ESP-Now, Thoracic biomechanics

## Abstract

Wearable respiration monitors often struggle with motion artifacts and variable skin–sensor coupling. We present an elastic chest belt with digitally printed piezoresistive strain gauges and a multi-channel bridge front-end, and we evaluate how sensor geometry, placement, and simple respiration cycle detectors affect minute-scale respiration-rate (RR) accuracy. Three printed sensor layouts (circular and serpentine/“linear”) were placed at three thoracic locations (left, center, right); for the serpentine layout, we also tested horizontal vs. vertical orientations. RR from (i) a time-domain peaks-and-valleys (PV) detector and (ii) a power spectral density (PSD) method were compared to a thermistor reference across 4-min trials in healthy volunteers (*n* = 3), analyzed per minute. PV achieved a larger share of minutes within ± 2 RPM than PSD (≈ 70% vs. ≈37%) and within ± 1 RPM (≈ 44% vs. ≈33%). Geometry and placement mattered: a vertical serpentine on the lateral thorax showed the highest minute-level agreement within this pilot cohort and under quiet-breathing conditions (left-lateral vertical: 100% of minutes within ± 2 RPM; right-lateral vertical/horizontal: 91.7%), while circular sensors showed their highest accuracy on the left thorax (83.3% within ± 2 RPM). These pilot findings suggest preliminary design guidance for e-textile RR monitors and indicate that PV detection may be a practical option for minute-scale RR estimation under the tested conditions.

**Supplementary Information:**

The online version contains supplementary material available at 10.1007/s44397-026-00054-0.

## Introduction

Respiration rate (RR) is a core vital sign linked to clinical deterioration and patient triage [1, 2], yet it remains difficult to monitor unobtrusively in daily life [3, 4]. Conventional airflow sensors and inductive/plethysmographic belts can be accurate, but their bulk, motion sensitivity, and need for rigid housings or tight straps limit long term comfort and adherence outside of controlled settings [3, 5, 6]. By contrast, soft, textile integrated strain sensors promise conformal, body compliant monitoring that can be worn continuously with minimal user burden [7–11].

The rapid evolution of printed and stretchable electronics has enabled this shift [12]. Additive patterning of conductors, semiconductors, and dielectrics onto elastomers and textiles [7, 13]—via direct ink writing, screen and stencil printing, aerosol or spray deposition, and transfer printing [14–21]—yields thin, flexible devices that survive the strains of the human body [22]. Progress spans materials (conductive hydrogels [23–26] and iono/organogels [27–29]; recyclable and reprocessable conductors [30–36]; high conductivity and biocompatible conductive inks [28, 35, 37–39]; electroactive materials [40–44]; elastomeric encapsulants and interconnects [45–49], components (dry skin electrodes for electrophysiology [50–57]; soft pressure and strain sensors [58–71]; flexible batteries and power modules [72–74], and systems (soft printed electronics for augmented human sensing [75] or full wearables for patient monitoring [76–81]. We build on this ecosystem to target robust respiration monitoring.

Within health monitoring, electrophysiology wearables have shown how mechanical and electrical transduction can be engineered for high quality signals on moving, sweating, and stretching skin. Lessons from epidermal ECG, EMG, and EEG—such as skin safe interfaces, high conformance contact, patch structure and fabrication, and dedicated low power, small size front ends [31, 50, 51, 53, 58, 59]—translate directly to mechanical sensing of the thorax. However, RR sensing from chest motion presents distinct challenges: thoracic strain fields are anisotropic and position dependent across ribs, sternum, and abdomen [82]; belt tension and posture modulate coupling [83]; and simple frequency domain estimators are often brittle to non stationarities such as sighs, speech, or brief movements [84, 85]. As a result, many wearable RR systems perform well in the lab but degrade in daily use [82, 83, 85].

In the broader state of the art for respiration health monitoring, instrumentation spans signals that co vary with breathing—airflow and gas composition at the airway [4, 5], chest and abdominal motion driven by thoraco abdominal biomechanics [6, 82], and slower changes in transcutaneous gas exchange [86–88]. Invasive or semi invasive approaches such as transcutaneous carbon dioxide monitoring rely on a heated electrode applied to the skin to enhance diffusion and estimate CO2 changes continuously. These devices are valuable in critical care and neonatal monitoring but do not directly measure RR, require heat at the skin that can irritate fragile tissue, and are not designed for comfortable day long wear outside clinical supervision [88–91].

Noninvasive techniques dominate outside the clinic. Airflow based methods position thermistors, thermopiles, humidity sensors, or capnography cannulas at the nose and mouth to exploit the temperature and composition difference between inhaled and exhaled air [92–95]. They can be accurate but are intrusive, can alter natural breathing, and add dead space that is undesirable for long term or free living use [95–99]. The other major family senses chest and abdominal motion. Respiratory inductance plethysmography (RIP) places wire coils in elastic bands around the thorax and abdomen and demodulates changes in inductance as the body expands and contracts [95, 100, 101]. RIP is mature and well validated, yet it requires careful placement and calibration, its oscillators and demodulators add complexity and power draw, and the coils are susceptible to electromagnetic interference and deformation [96, 101–105].

Skin conforming and textile integrated motion sensors avoid airway instrumentation and can be embedded in garments. Among stretchable transduction modes, inductive, capacitive, and resistive strategies have all been explored [93, 101, 103, 106, 107]. Inductive systems map changes in belt geometry to shifts in resonance frequency; they are contactless in principle but require stable oscillators, careful shielding, and mechanically robust coil layouts. Capacitive systems build a deformable capacitor from compliant electrodes and a soft dielectric; their signal is strong but depends on fit, suffers from stray capacitances from sweat and posture, and typically needs driven shields and stable excitation. Resistive or piezoresistive systems exploit the change in conductor geometry and percolation under stretch, producing a simple resistance change that is easily read with a Wheatstone bridge and modest instrumentation gain. Resistive elements are straightforward to deposit directly onto textiles by printing or coating, support arbitrary geometries that can be aligned with anatomic strain fields, and are cost and power efficient; their weaknesses—thermal drift, hysteresis, and motion artifacts—can be mitigated with filtering, thoughtful placement, and robust algorithms [93].

Taken together, the state of the art points to a pragmatic rationale for the present work. For garment scale, everyday monitoring, resistive strain sensing offers the best match to manufacturability, cost, and low power electronics while preserving flexibility in geometry and placement. We therefore adopt printed, resistive transducers and focus on the engineering choices that matter most in practice: the orientation and geometry of the traces relative to dominant thoracic strain, the placement of sensors across the lateral ribs and midline, and the selection of an RR estimator that tolerates non stationarity without heavy computation.

Printed, textile integrated strain sensors offer a compelling path if we choose where to sense, how to orient the transducers relative to dominant strain vectors, and what algorithm to use for minute scale RR estimation on resource constrained hardware. These choices are rarely compared head to head, and guidance is scattered across studies that differ in materials, electronics, and evaluation protocols. There is a practical need for comparative and empirically grounded design guidance that maps geometry, placement, and signal processing choices to achievable accuracy that matters for wearables.

**Fig. 1:**
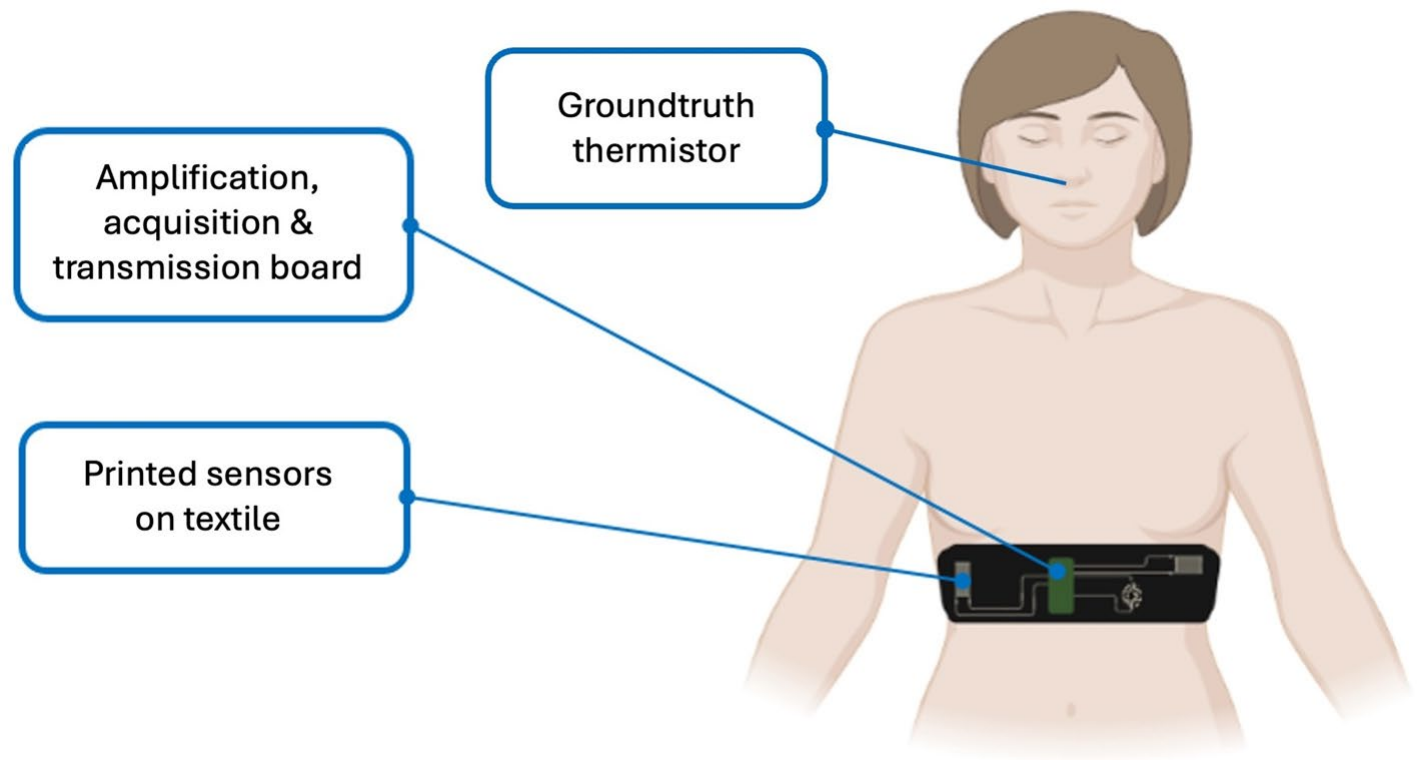
Wearable respiration-monitoring platform used in this study. Digitally printed piezoresistive strain sensors are integrated into an elastic textile belt and connected to a compact readout module that performs amplification, acquisition, and wireless transmission. A near-nostril thermistor serves as the ground-truth reference for respiration cycles during validation

This study addresses that need using a printed, multi-sensor chest belt, as shown in Fig. 1 and a pilot dataset (*n* = 3). We ask three questions: (1) Which sensor geometry and orientation yield the most reliable RR during quiet breathing? (2) Which thoracic placement (left, center, right) is most robust? (3) For minute-scale RR, does a simple peaks and valleys (PV) detector outperform a power spectral density (PSD) approach? We answer these with per minute accuracy statistics suited to wearable deployment and distill preliminary guidance for future e textile respiration monitors.

## Materials and methods

### Printed e-textile belt and sensors

A biphasic elastomer-metal ink (Ag-In-Ga-SIS) was prepared as follows: SIS (Styrene-Isoprene Copolymer –Aldrich Chemistry) is first diluted in Toluene (3:1 SIS: Toluene wt%). Silver flakes (Ag 071 Silflake, Technic) are the mixed into the SIS solution (2:1 wt%) using a planetary mixer (Thinky ARE-250) for 3 min at 2000 rpm. A pre-made Eutectic Galium-Indium mixture (EGaIn; 75.5% Ga, 24.5% In), is mixed into the Ag-SIS solution (2:1 EGaIn: Ag wt%) for 3 min at 2000 rpm. The resulting Ag-In-Ga-SIS, which presents a shiny silvery color is loaded into a syringe barrel for further printing. Schematic details on the ink fabrication process are shown in Support Information Figure S1. The ink’s phase structure and electromechanical characterization have been previously published in refs. [50, 108].

First, the standalone strain gauges were direct-ink-written (DIW) using a commercial ink extrusion printer (Voltera V1, Voltera) onto TPU film (3412 TPU hotmelt film, Bemis) following digitally designed traces. Two layers of ink are dispensed on top of each other to reduce sheet resistance, and the print is then cured at room temperature for 2 h. This method was used to print the stain gauges since it allows for better printing resolution than other printing methods, but at a cost of a small printing area.

**Fig. 2:**
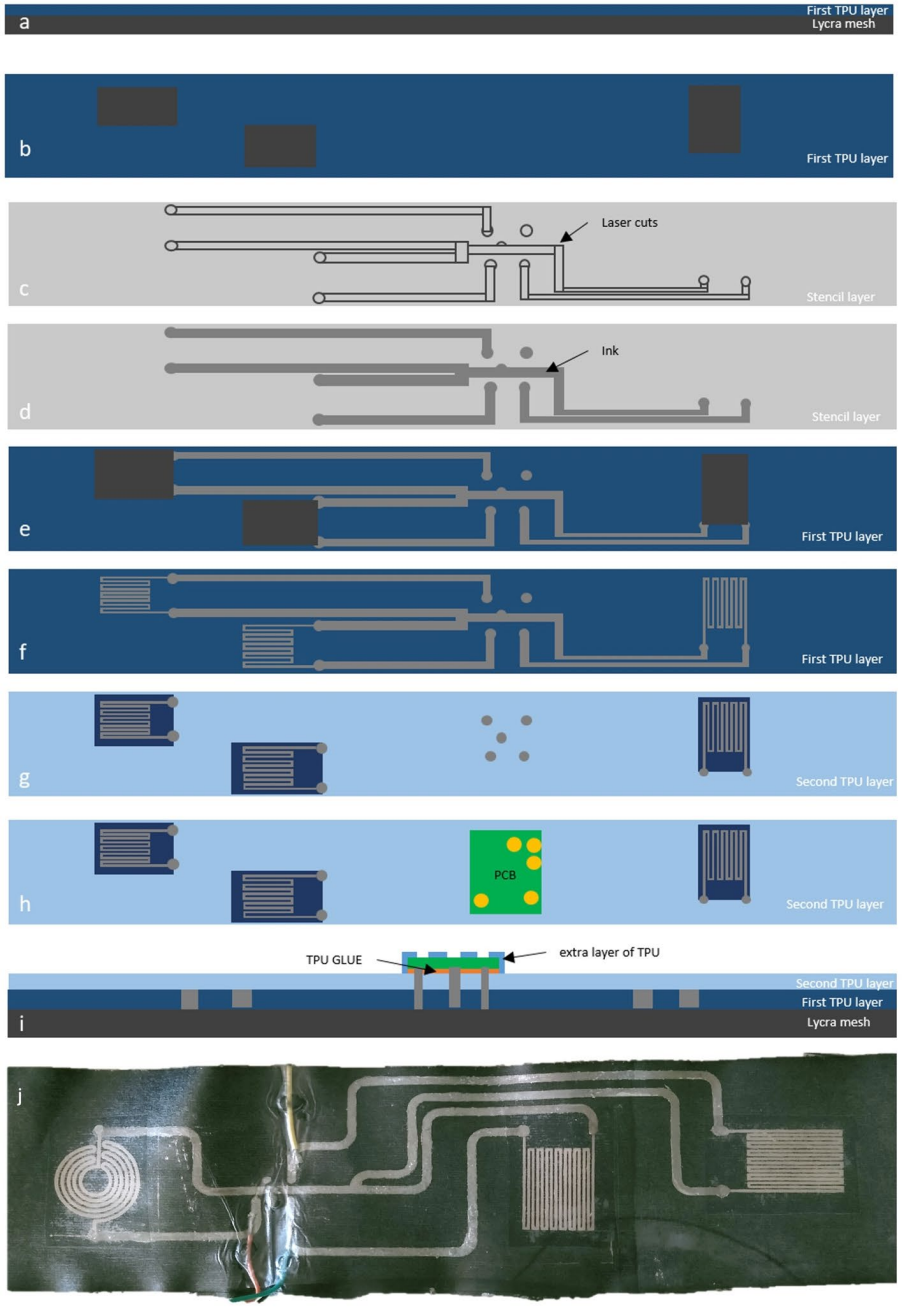
Fabrication workflow of the textile-printed respiration belt. **a** Laminate a first thermoplastic polyurethane (TPU) film onto 4-way Lycra. **b** Add TPU “islands” to define mechanically reinforced zones for sensor pads and the electronics. **c** Laser-cut a stencil with the interconnect and pad geometry. **d** Stencil-print the conductive ink (Ag–In–Ga–SIS) through the mask to form tracks and contact pads; remove the mask after curing. **e**, **f** Print the sensing elements on TPU - circular and serpentine layouts - and heatpress them to the interconnects; heat-laminate the printed sheet to the base layer. **g**, **h** Laminate a second TPU film as an encapsulant, with openings for vias/connector pads, and mount the readout PCB onto the contact pads. **i** Cross-section of the final stack showing Lycra substrate, first TPU, printed conductors with TPU adhesive, and the second TPU encapsulant (with an extra TPU layer over the electronics). **j** Photograph a completed belt with circular and serpentine strain sensors and routed interconnects

For the wider lines that connect the printed strain gauges transducers to the rigid board, a stencil printing process is employed. First, TPU ST604 film is heat pressed on top of an elastic lycra band. It is relevant to note that the TPU film doesn’t cover the portions of the textile to where the printed transducers will be later transferred, Fig. 2a, b. With the belt substrate ready, the stencil for the printed interconnects is laser cut, as shown in Fig. 2c. After the laser patterning step, the stencil is placed over the TPU-Lycra stack, and a layer of conductive Ag-In-Ga-SIS ink then spread evenly throughout the belt to print the cut pattern as shown in Fig. 2d. Right after filling the pattern with the conductive ink, the stencil is carefully removed, as depicted in Fig. 2e, and the ink is let to cure/dry for 2 h at room temperature (~ 23 °C, no humidity control).

We then make sure that there are no shorts in the circuit by testing continuity across the printed lines. Worth noting that no shorts were found in any of the fabricated belts. The previously DIW strain sensors are then heatpressed into to the chest band in the spaces that were previously left without TPU, as shown in Fig. 2f.

An extra layer of TPU is then bonded on top on the full belt so the conductive traces become fully encapsulated. The strain gauges are left without encapsulation, as this would affect their electromechanical response due to the viscoelastic nature of the encapsulant. The encapsulant layer also leaves the pads for interfacing with the rigid acquisition board uncovered, as shown in Fig. 2g–i. Velcro (hook and loop) fabric is finally glued to each side of the lycra (spandex) band to secure it around the body. The full printed belt is shown in Fig. 2j.

All sensors were printed with the same Ag–In–Ga–SIS ink and identical process parameters, thus we expect no difference in the piezoresistivity of the ink across designs. The strain-induced resistance modulation during breathing depends on geometry because each layout samples different components of the in-plane strain field. For small deformations, the relative resistance change can be expressed conceptually as ΔR/R₀ ≈ GF·ε_eff_, where GF is the material gauge factor and ε_eff_ is the strain component effectively projected along the conductor path (a weighted average over segments in multi-turn traces). Thus, orientation and layout primarily change ε_eff_ and the signal-to-noise ratio rather than the intrinsic material response.

The various sensor shapes and orientations are discussed in further sections.

### Rigid acquisition circuit

**Fig. 3:**
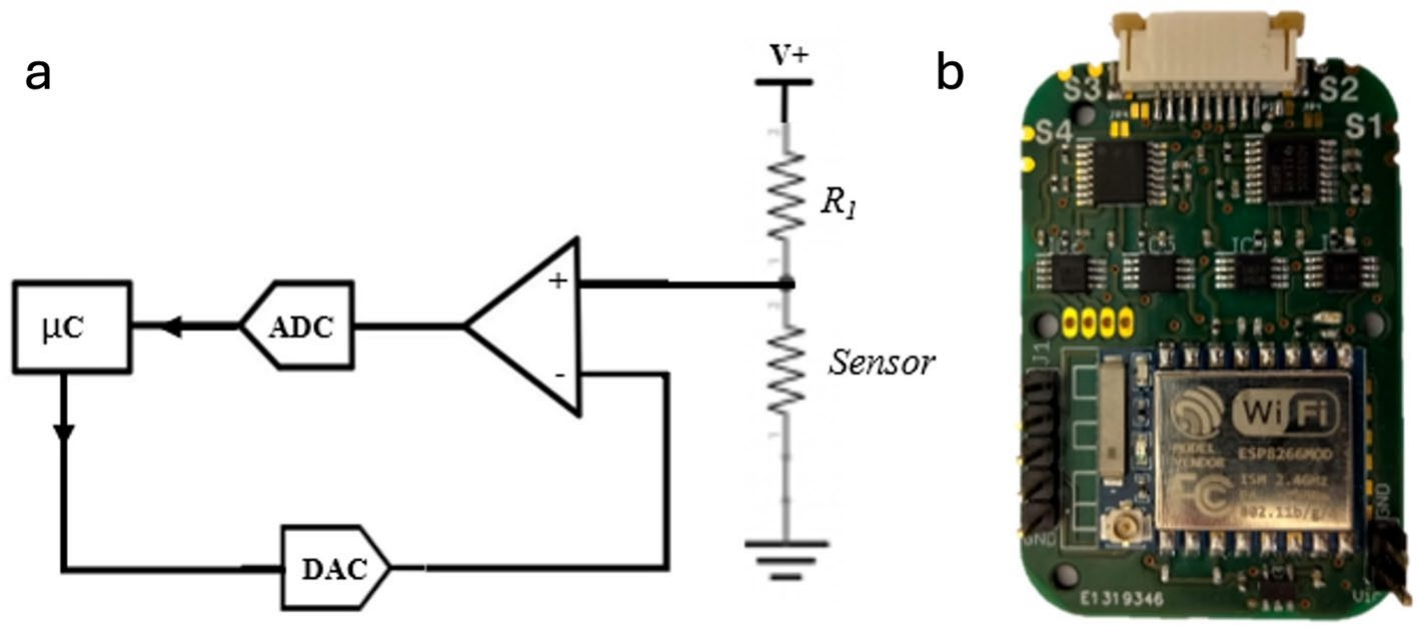
Readout electronics and acquisition module. **a** Simplified block diagram of one channel: the printed strain element (R₁) is conditioned by a differential stage with DAC-based offset cancellation and digitized by a 24 bit ADC (PGA gain = 1); a DAC under microcontroller (µC) control injects an active offset to center the signal before digitization by the ADC, avoiding saturation. **b** Photograph of the custom four-channel board integrating the analog front end, ADC, and an ESP-based wireless microcontroller, with connectors (S1–S4) for the textile sensors

To acquire the signals from the printed resistive sensors, we developed a circuit to automatically remove DC offset from the output of a single voltage divider, as depicted in Fig. 3a. For this, we used a digital to analog converter (DAC) controlled by the microcontroller unit. The DAC signal and sensor output are fed into a differential amplifier stage so that DC offset is removed to prevent saturation of the system. In the prototype used in this study, digitization was performed using an ADS122C04 (24‑bit ΔΣ ADC) configured with the internal 2.048 V reference and PGA gain = 1. The offset-injection stage used a DAC8571 (16‑bit DAC). The DAC output is adjusted during a short auto‑zero routine at the start of each acquisition to cancel the DC offset (center the signal) and is then kept constant during the subsequent recording/1‑min analysis windows.

A printed circuit board (Fig. 3b) integrating 4 of these “self-calibrating” acquisition stages was developed, while also integrating a ESP8266 module for simultaneous acquisition of up to four resistive sensors and real-time transmission of the data via Wi-Fi (ESP-Now protocol) to a remote host for logging and further processing. For our tests, we used 3 printed sensors, and a thermistor (resistive temperature sensor also connected to one of the four channels of the PCB), placed near the user’s nostril, to serve as the respiration groundtruth. Since all signals (thermistor and strain sensor) were connected to the same acquisition circuit, they were sampled simulatenously avoiding the need for further signal alignment.

The exposed ENIG pads on the bottom of the board (identified as S1-4 in Fig. 3b), allow for seamless connection to the printed lines on the elastic band as described earlier.

### Signal preparation and filtering

To better identify the respiration rate, a first approach is to filter out the motion artifacts or other types of noise that occur at specific frequencies. Having in mind that the respiration rate is between 0.2 and 1.8 Hz, a band pass filter from 0.1 to 2 Hz is enough to capture the respiration signal without losing any of its important components [109].

After band-pass filtering (0.1–2 Hz), signals were smoothed using a Savitzky–Golay filter (window length 21 samples, polynomial order 0, derivative order 0) to reduce residual high-frequency fluctuations while preserving respiratory-cycle timing. The preprocessing chain was fixed and applied identically to all subjects and configurations. The Savitzky-Golay smoothing and differentiation filter can be used to reduce high frequency noise in a signal due to its smoothing properties and reduce low frequency signal using differentiation. These properties are the reason the “savgol” filter is one of the most popular signal processing tools in spectroscopy and chemometrics. Additionally, weighted least-squares can provide more control over the design of the savgol filter.

The savgol filter is used to smooth the signal around the peaks and valleys of the signal in order to have a better clean signal. For a given signal measured at N points and a filter of width, w, savgol calculates a polynomial fit of order 0 (corresponding to a moving average), with this the noise around the peaks and valleys that was not filter previously can be mitigated.

The same signal filtering pipeline was used for both the printed strain sensors and baseline thermistor.

### Algorithms for respiration rate detection

**Fig. 4:**
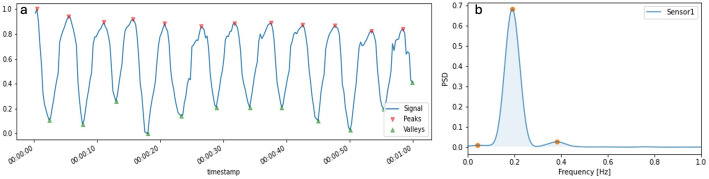
Respiration-rate extraction from a printed strain sensor using the two estimators compared in this work. **a** Band-pass–filtered (0.1–2 Hz) and smoothed strain signal over a 60-s window with detected peaks (red) and valleys (green); RR is computed from the median peak-to-peak (or valley-to-valley) interval within the 60‑s window. **b** The corresponding power spectral density (PSD) for the same segment; RR is obtained from the dominant spectral peak after conversion to breaths per minute

To acquire the Respiration Rate(RR), two algorithms where used: Peaks and Valleys (PV) and Power Spectral Density(PSD). The first one -PV algorithm, exemplified in Fig. 4a- searches for variations in the signal, looking for its peaks and valleys independently. The function considers that a peak exists when the previous sample value is lower than the value of current sample, ilocs_min. For finding the valleys, the approach is similar: the algorithm finds where when the previous sample value is larger than the value of the current sample, ilocs_max.

Peaks and valleys were detected using “argrelextrema” (Python function) with neighborhood parameter order = 10 samples. No additional amplitude thresholding or hysteresis was used. Spurious detections were rejected by constraining consecutive peak-to-peak (or valley-to-valley) intervals to the physiological range implied by the passband (0.5–10 s). RR per 60‑s window was computed as RR = 60/median(Δt) from the valid inter-peak intervals (and analogously for valleys).

The power spectral density (PSD) function, on the other hand, shows the strength of the signal variations(energy) as a function of frequency. In other words, it shows at which frequencies variations are strong and at which frequencies variations are weak. With that in mind, with the PSD is possible to see what the most relevant frequencies of the signal are and acquire the RR. “PSD was computed per 60‑s window using a conventional Welch estimator (Hann window, 60‑s segment, no overlap). The dominant peak within 0.1–2 Hz was selected and converted to breaths/min (RR = 60·f). The unit of PSD is energy (variance) per frequency(width) and one can obtain energy within a specific frequency range by integrating PSD within that frequency range. The outcome of the PSD is shown in Fig. 4b.

We implemented PV and a simple PSD-based baseline to compare a time-domain cycle-counting approach against a conventional frequency-domain dominant-peak estimate under the same 60‑s analysis window. This comparison is intended as an engineering baseline under the tested conditions, not a general ranking of time- vs. frequency-domain methods.

### Participants and test protocol

**Fig. 5:**
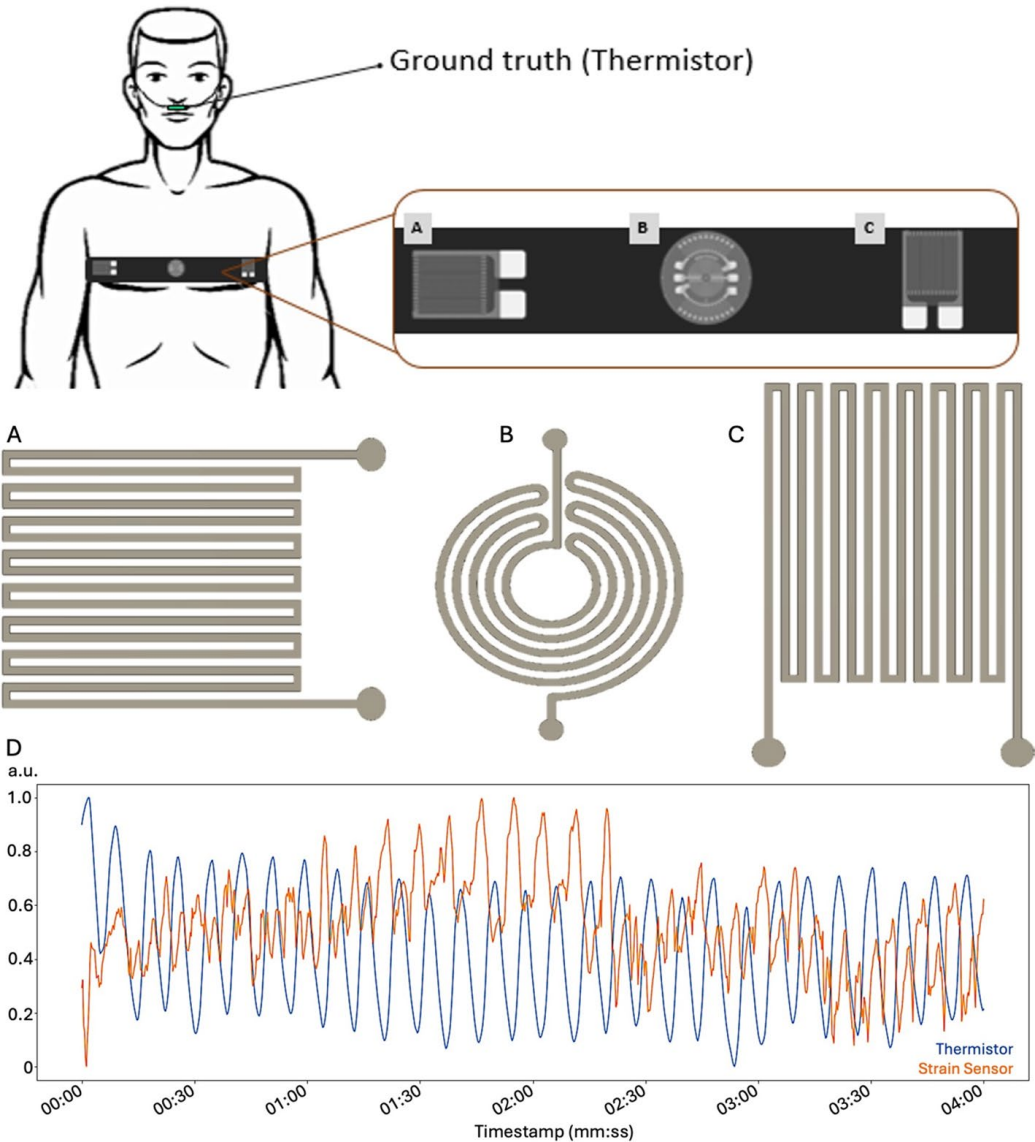
Experimental setup and printed-sensor geometries used in the placement/orientation study. The elastic belt is worn across the upper thorax while a near-nostril thermistor provides the ground-truth respiration reference. The instrumented belt hosts three printed strain sensors: **A** (left) a linear serpentine oriented horizontally, **B** (center) a circular spiral, and **C** (right) a linear serpentine oriented vertically. The drawings show the three geometries as fabricated on TPU prior to lamination. **D** representative quiet-breathing segment illustrating synchronous cycles from the thermistor (blue) and a printed strain gauge channel (orange)

Healthy adult volunteers (*n* = 3) performed 4-minute trials. Chest circumference/body habitus was not used as a stratification variable in this pilot study, and trials were limited to quiet breathing (no paced shallow/deep/irregular breathing protocol). The age, sex, and weight distributions are shown in Table 1. A bead thermistor placed near the nostrils served as the ground-truth for respiration cycle counts, as shown in Fig. 5. In each test we used the same respiration rate detection algorithm for the nostril thermistor and the printed sensor.

**Fig. 6:**
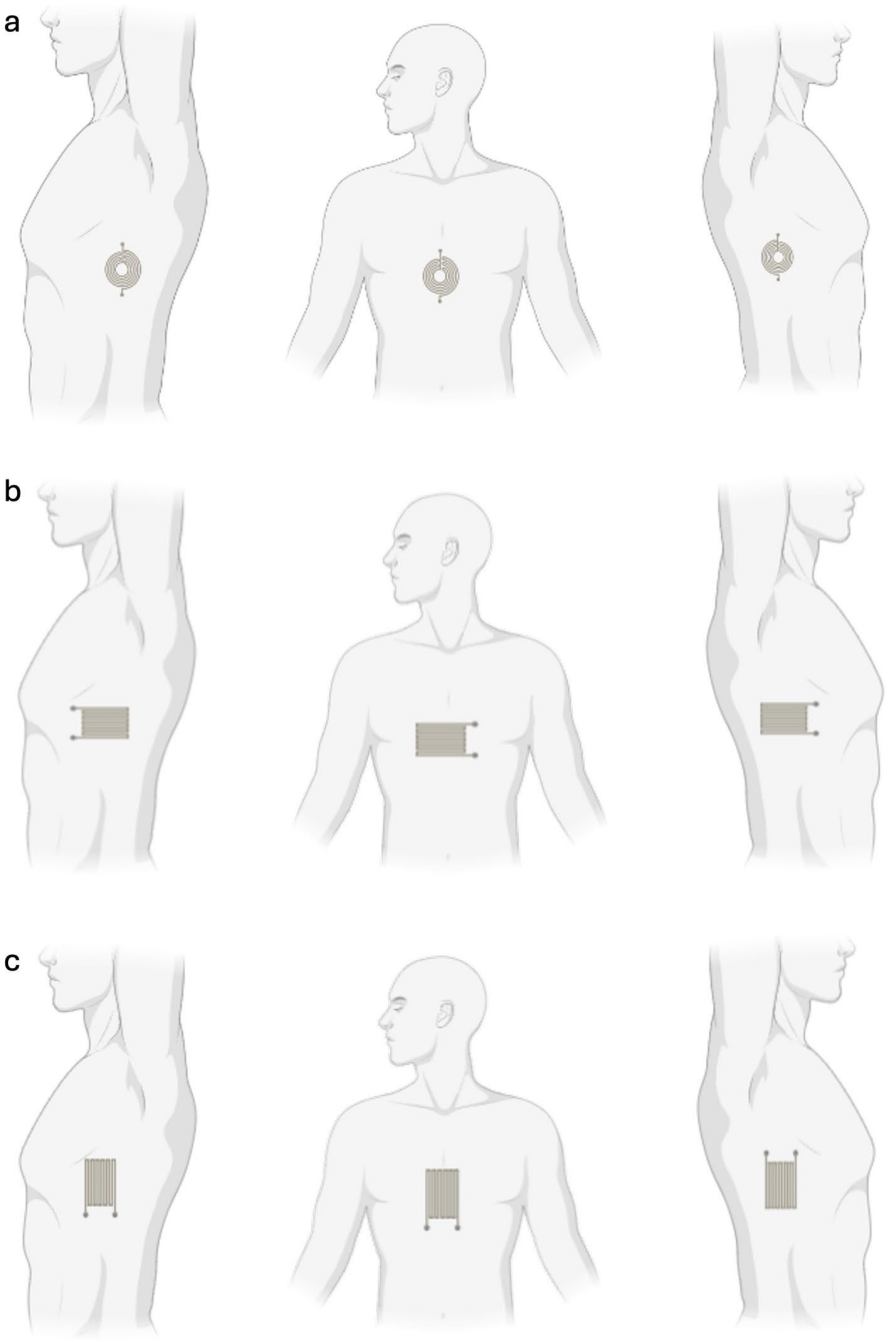
Sensor placement matrix for the thoracic study. **a** Circular (orientation-agnostic) strain sensor positioned at the left lateral ribs, midline (sternum/upper abdomen), and right lateral ribs. **b** Linear serpentine horizontal orientation at the same three positions. **c** Linear serpentine vertical orientation at the same three positions. All sensors are printed on TPU and laminated onto the elastic belt; drawings not to scale

For each printed geometry (radial and longitudinal strain gauges, shown in Fig. 5A, B), sensors were positioned at three thoracic locations—left lateral ribs, center sternum/upper abdomen, and right lateral ribs, with horizontal and vertical orientations tested for the longitudinal layout (Fig. 5A, C). A representative example of the signals acquired from the thermistor and a strain sensor is shown in Fig. 5D. All tested sensor orientations and positions in the body are shown in Fig. 6.

All trials for the same sensor position/orientation used the same belt, while all tests used the same belt design (same elastic textile/TPU stack and Velcro closure, etc.); belt tension was adjusted to a snug, comfortable fit for each subject, but belt material/elasticity was not varied as an experimental factor.

Signals were analyzed in non-overlapping 1-minute segments. Each 4‑min trial therefore yields four non-overlapping 60‑s windows per subject. Unless otherwise stated, accuracy percentages and MAE values are reported at the window level and were computed by pooling all 1‑min windows across subjects for each configuration (i.e., not computed per subject and then averaged). As a consequence, the window counts represent repeated measures within subjects and should be interpreted as descriptive statistics in this pilot cohort (*n* = 3).

Because RR is computed from cycle counts in fixed 60‑s windows, the ± 1 and ± 2 RPM thresholds are reported here as descriptive engineering error bands (equivalent to miscounting by 1 or 2 breaths per window) for comparative purposes, and are not intended as clinical acceptability criteria.

**Table 1:** Participant demographics

	Sex	Age (Y)	Weight (Kg)
Subject 1	Male	23	81
Subject 2	Female	29	68
Subject 3	Male	54	92

## Results

### Peaks and valleys vs. power spectral density

**Fig. 7:**
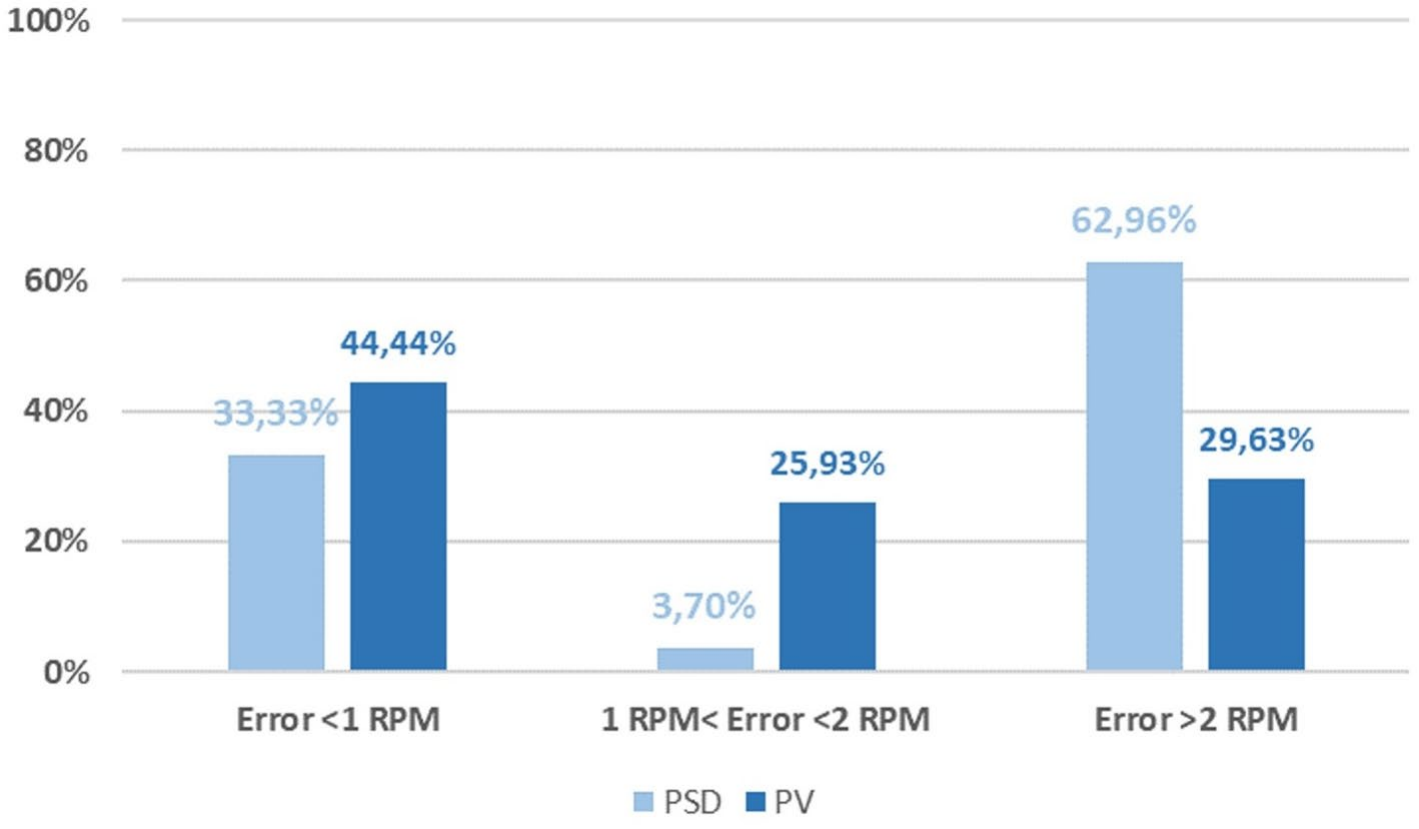
Minute-level respiration-rate error distribution for PV and PSD (pooled across non-overlapping 1‑min windows). Bars show the share of windows in the three error bins (< 1 RPM, 1–2 RPM, > 2 RPM). Summary accuracy within ± 1 and ± 2 RPM is reported in Table 2

Across all subjects and configurations, we evaluated RR error in non-overlapping 1‑min windows and computed summary statistics by pooling windows across subjects (pilot cohort *n* = 3). Given nine configurations per subject and 4‑min trials, this corresponds to 108 1‑min windows in total (3 subjects × 9 trials × 4 windows), which should be interpreted as repeated measures rather than independent samples. Under these conditions, PV yielded a higher share of accurate windows than PSD. PV yielded higher minute-level agreement than PSD (Table 2), and Fig. 7 shows the corresponding error-bin distribution across windows. This suggests PV is less sensitive to brief non-stationarities(sighs, posture shifts) than PSD under the tested quiet-breathing protocol.

**Table 2:** Accuracy by algorithm (percentage of 1‑min windows)

Algorithm	Error ≤ ± 1 RPM	Error ≤ ± 2 RPM
Peaks‑and‑Valleys (PV)	44.44%	70.33%
Power Spectral Density (PSD)	33.33%	37.03%

The PSD analysis provides a complementary view of signal frequency content; however, the dominant-peak PSD baseline used here can be sensitive to windowing choices and short transients in minute-scale, non-stationary traces. In comparison, the peaks and valleys algorithm yielded higher minute-level agreement than PSD in capturing temporal dynamics and accurately quantifying respiratory rate in this pilot dataset. Based on this pilot comparison, we used PV for the subsequent analyses because it provided higher minute-level agreement under the tested quiet-breathing protocol. Having these results into account, the following results will be presented using the Peaks and Valley algorithm. This should not be interpreted as a general limitation of frequency-domain methods, as more advanced spectral/time–frequency estimators may perform differently.

The Supporting Information provides subject-level RR estimates and absolute errors for each configuration (Tables S1–S6) alongside representative raw traces (Figures S1–S55), enabling inspection of inter-subject variability.

### Geometry and placement - circular sensor

**Fig. 8:**
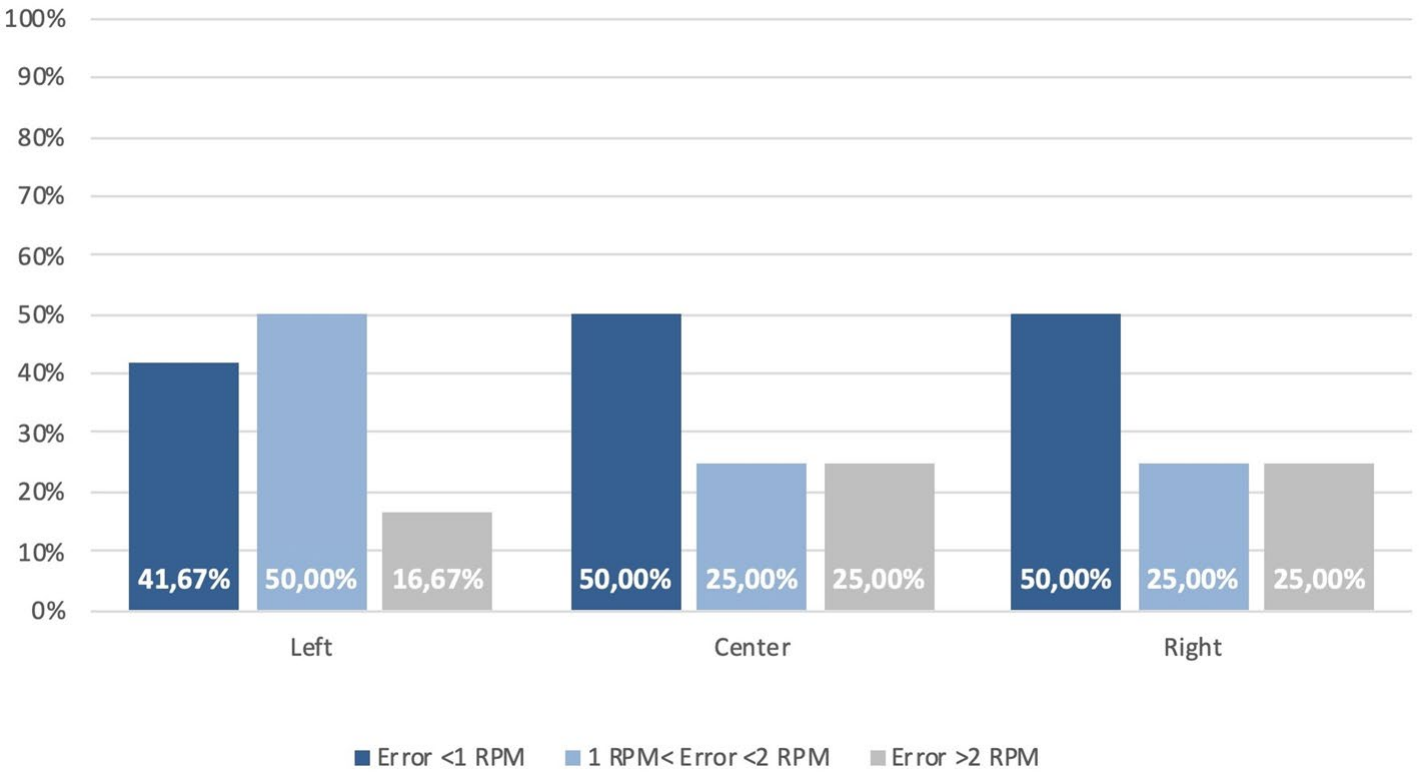
Circular sensor—minute-level RR error distribution by thoracic position using PV (pooled across subjects; non-overlapping 1‑min windows). Bars show the share of windows in < 1 RPM, 1–2 RPM, and > 2 RPM error bins. Summary accuracy within ± 1 and ± 2 RPM is given in Table 3

The circular transducer is orientation‑agnostic owing to its radial symmetry, so this experiment isolates placement as the sole design variable. Using the PV estimator (§ 3.1) and pooling all recordings across participants, without considering subject-level inference (*n* = 12 1‑min windows per placement), Fig. 8 summarizes the proportion of analysis windows meeting the ± 1 and ± 2 RPM criteria for placements at the left lateral thorax, center (sternum/upper abdomen), and right lateral thorax; the corresponding percentages are listed in Table 3. The left placement yielded the highest accuracy within this pilot dataset, with 83.33% of windows within ± 2 RPM and 41.67% within ± 1 RPM. The center and right placements both reach 75.00% within ± 2 RPM and 50.00% within ± 1 RPM, indicating broadly comparable performance between these two sites.

Two qualitative observations from the raw traces (shown per subject in the Supplementary Material) help explain these differences. First, center placement exhibits higher baseline wander and more frequent low‑amplitude oscillations superimposed on the breathing rhythm, likely reflecting abdominal contributions and belt micro‑slip over soft tissue. Second, the right placement behaves similarly to the left but shows slightly more susceptibility to transient over‑counts when the belt is adjusted or during brief posture changes. Because the circular geometry cannot be aligned to the dominant strain vector, its coupling to the rib‑cage motion depends strongly on local anatomy and belt tension; lateral placements, particularly on the left, appear to provide the most repeatable coupling across our pilot cohort. While absolute percentages change if PSD is used instead of PV, the ranking by placement is preserved, reinforcing that placement - rather than algorithm choice - drives the outcome for this geometry.

From a garment‑design perspective, these pilot results suggest that a single circular sensor can be considered as a secondary channel placed laterally (preferably left) when orientation control is not desired or when symmetry with other sensors is required. If aesthetics or routing mandate a center placement, adding at least one lateral strain sensor (see § 3.3) may improve robustness.

**Table 3:** Circular sensor accuracy by position (percentage of 1‑min windows)

Position	Error ≤ ± 1 RPM	Error ≤ ± 2 RPM
Left	41.67%	83.33%
Center	50.00%	75.00%
Right	50.00%	75.00%

### Geometry, placement, and orientation—serpentine (“linear”) sensor

**Fig. 9:**
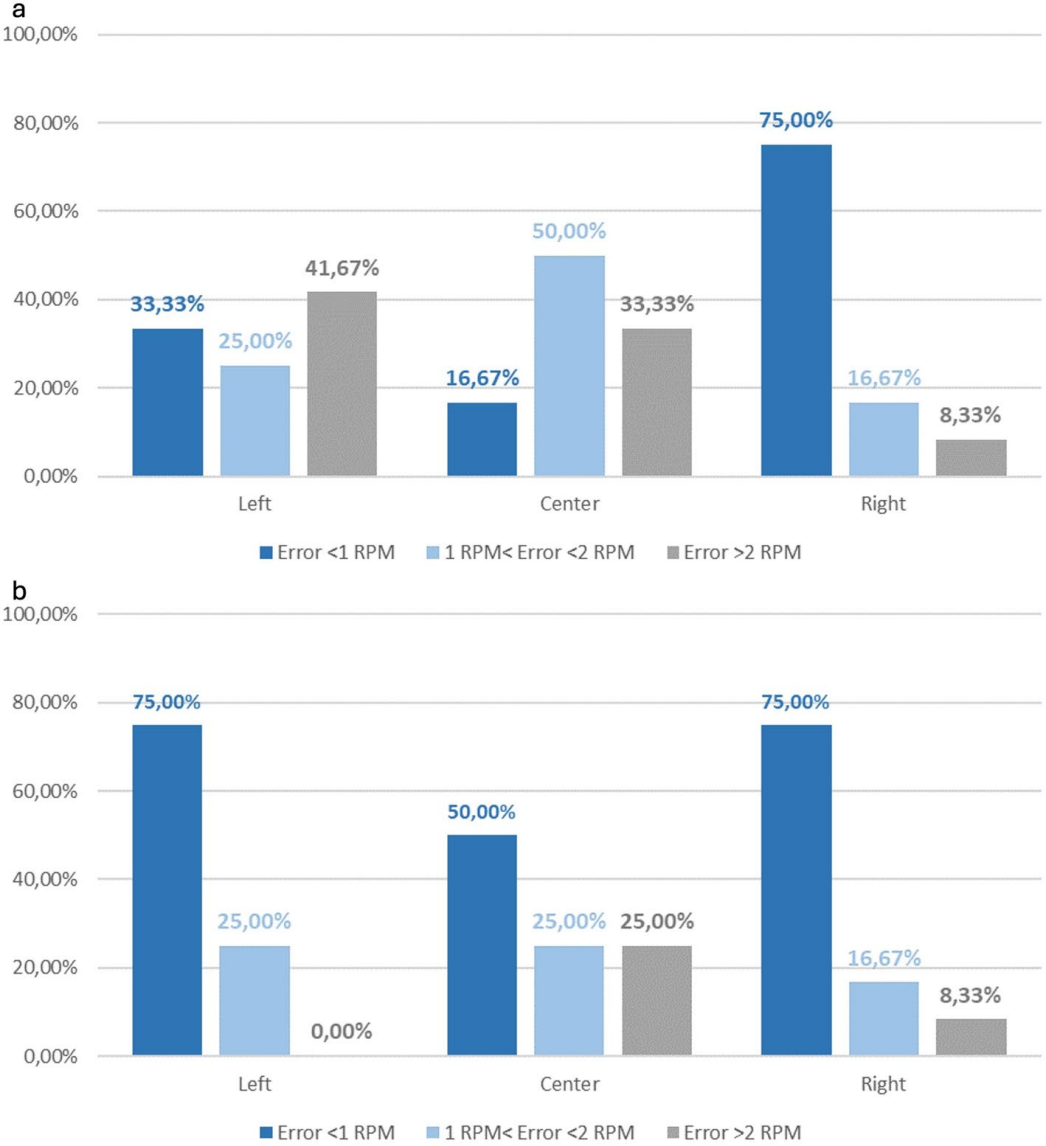
Serpentine sensor—effect of thoracic placement and trace orientation on minute-level RR accuracy using PV (pooled across subjects; non-overlapping 1‑min windows). **a** Horizontal orientation. **b** Vertical orientation. Bars show the share of windows in < 1 RPM, 1–2 RPM, and > 2 RPM error bins. Summary accuracy within ± 1 and ± 2 RPM is reported in Table 4

For the serpentine layout, orientation relative to the principal thoracic strain is pivotal. We again pool all recordings and use the PV estimator for comparability. Figure 9a reports the horizontal‑orientation results for left, center, and right placements; Fig. 9b reports the vertical‑orientation results for the same three positions. Table 4 consolidates the percentages.

Across placements, vertical orientation tended to yield higher accuracy than horizontal (Table 4; Fig. 9), with the largest gain at the left lateral site where vertical reached 100% within ± 2 RPM. Right-side performance was high for both orientations, and midline performance was lower overall.

Mechanically, these trends are consistent with thoracic biomechanics: vertical serpentines align with the superior–inferior expansion of the rib cage during quiet breathing and are less sensitive to skin shear and belt sag, whereas horizontal serpentines are loaded primarily in shear and bending and thus pick up posture‑dependent artifacts. The left‑vertical configuration yielded the highest accuracy in our pilot dataset, with the right‑vertical and right‑horizontal close behind. The center‑vertical placement is acceptable but less reliable, and center‑horizontal is the weakest configuration.

These results suggest a design direction. In our tested setup, placing vertical serpentines on the left and right lateral thorax may offer a favorable balance of accuracy and redundancy. A circular midline sensor can then serve as a backup. Notably, repeating the analysis with PSD lowers absolute accuracies but preserves the same ordering of configurations, underscoring that geometry and placement dominate over algorithm choice for the serpentine layout.

**Table 4:** Serpentine sensor accuracy by position and orientation (percentage of 1‑min windows)

Position	Orientation	Error ≤ ± 1 RPM	Error ≤ ± 2 RPM
Left	Horizontal	33.33%	58.33%
Left	Vertical	75.00%	100%
Center	Horizontal	16.67%	66.67%
Center	Vertical	50.00%	75.00%
Right	Horizontal	75.00%	91.67%
Right	Vertical	75.00%	91.67%

### Overall ranking and belt design choice

**Fig. 10:**
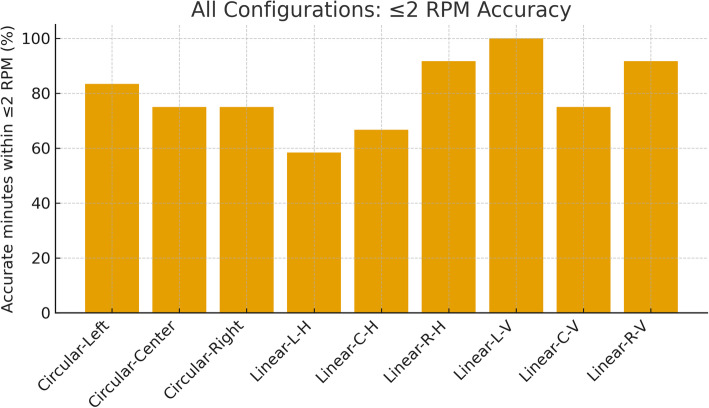
Overall accuracy across the nine sensor–placement configurations, expressed as the percentage of 1‑min windows within ± 2 RPM (PV estimator; pooled across subjects)

To compare all geometries and placements at a glance, Fig. 10 ranks the nine configurations by the share of windows within ± 2 RPM. The linear left‑vertical configuration ranked highest in this pilot dataset at 100%, followed by linear right‑vertical and linear right‑horizontal (both 91.67%). The circular left reaches 83.33%, while circular center and circular right sit at 75.00%. The lowest-performing configurations in this dataset are the linear left‑horizontal (58.33%) and linear center‑horizontal (66.67%). These rankings are robust to the choice of estimator: using PSD lowers absolute percentages but preserves the ordering, indicating that geometry and placement, not the algorithm, dominate outcomes.

Beyond the ≤ 2 RPM metric, the distribution of errors and the mean absolute error (MAE) validate the same picture and help set design thresholds.

Table 5 summarizes the error-bin distribution and MAE for all configurations. The same hierarchy emerges: the left-vertical serpentine shows the lowest MAE and no > 2 RPM windows, whereas left-horizontal and center-horizontal serpentines show the largest error tails and may be less suitable as primary channels under the tested conditions. Circular sensors perform best when placed laterally.

Two practical takeaways follow. First, within this pilot dataset, a single-sensor design would likely benefit from a vertical serpentine on the left lateral thorax, as it combined zero > 2 RPM windows with the lowest MAE under our quiet-breathing protocol. Second, for a two‑sensor belt, deploy vertical serpentines on both lateral sides; this gives redundant high‑quality channels while maintaining garment symmetry. A circular midline sensor can be retained as a secondary channel for cross‑checking or fallback, but midline linear sensors—particularly horizontal— showed lower agreement in our pilot dataset and may be more sensitive to coupling and abdominal-motion artifacts under the tested conditions in our pooled dataset.

**Table 5:** Configuration-wise error distribution and MAE for RR estimates using PV (pooled across all recordings)

Sensor	Position/Orientation	Error < 1 RPM	1 RPM < Error < 2 RPM	Error > 2 RPM	Mean Error	Std Error
Circular	Left	41.67%	41.67%	16.67%	1.13	0.32
Linear	Left/Vertical	75.00%	25.00%	0%	0.65	0.12
Linear	Left/Horizontal	33.33%	25.00%	41.67%	1.93	0.54
Circular	Center	50.00%	25.00%	25.00%	1.21	0.27
Linear	Center/Vertical	50.00%	25.00%	25.00%	1.18	0.22
Linear	Center/Horizontal	16.67%	50.00%	33.33%	2.06	0.45
Circular	Right	50.00%	25.00%	25.00%	1.48	0.35
Linear	Right/Vertical	75.00%	16.67%	8.33%	0.93	0.39
Linear	Right/Horizontal	75.00%	16.67%	8.33%	0.97	0.40

The table reports the share of windows with Error < 1 RPM, 1–2 RPM, and > 2 RPM, along with MAE and standard error across windows

## Discussion and conclusion

We set out to answer three pragmatic questions that matter for a garment one actually wears: which printed geometry and placement deliver reliable respiration rate (RR), whether a simple time‑domain cycle counter can beat a frequency‑domain estimator, and how these choices translate into a low‑power system. The evidence from our pilot is coherent. In this pilot dataset, the PV detector yielded higher minute-level agreement than the simple dominant-peak PSD baseline implemented here, and the dominant lever on accuracy was not the algorithm but where and how the strain sensors were placed. In particular, a vertical serpentine positioned on the lateral thorax consistently outperformed alternatives, while midline placements and horizontal traces were more vulnerable to abdominal motion and belt slippage; circular traces were serviceable when placed laterally, but less reliable at the center.

These outcomes make sense when viewed through the mechanics of quiet breathing. Thoracic expansion is anisotropic: the lateral ribs move largely along a superior–inferior axis, whereas the midline mixes rib‑cage and abdominal excursions. A vertical serpentine aligns with the principal stretch on the lateral chest and thus transduces respiration efficiently while rejecting shear‑dominated artifacts; a horizontal serpentine is loaded primarily in shear and bending and is consequently more sensitive to posture and micro‑slip. Circular traces, attractive because they are orientation‑agnostic, cannot be aligned to the dominant strain vector and therefore depend more on local anatomy and belt tension for coupling. This mechanical picture explains our observed hierarchy (lateral‑vertical > lateral‑horizontal ≈ circular‑lateral > center) and is consistent with prior experience in stretchable electronics where serpentine orientation and neutral‑axis routing govern signal stability under motion. This explains why vertical serpentines on the lateral thorax provide the most robust RR: they maximize ε_eff under the dominant superior–inferior expansion, while horizontal serpentines are more sensitive to shear/bending and circular spirals average over directions.

The algorithmic comparison reinforces a system‑level point: minute‑long windows of everyday breathing are not stationary. People sigh, speak, or adjust posture; amplitudes drift with belt tension and thoraco‑abdominal contributions. In our dominant-peak PSD baseline, the method collapses these dynamics into a single dominant frequency, which can be biased by short transients and spectral leakage. PV instead treats respiration as a sequence of cycles, counting peaks and valleys after modest filtering and smoothing so that a transient perturbs only the affected cycles rather than the entire estimate. PV’s simplicity is also an engineering advantage: peak‑picking and basic quality checks execute comfortably on ultra‑low‑power MCUs, enabling on‑device computation and sparse, minute‑level telemetry. We emphasize that this comparison is limited to a conventional dominant-peak PSD implementation; adaptive spectral tracking or time–frequency methods may mitigate sensitivity to non-stationarity and are an important direction for future work.

Taken together, the results suggest preliminary design guidance for wearable RR under the tested quiet breathing conditions: instrument both lateral sides with vertical serpentines and keep a circular midline transducer as a secondary channel. Use a bridge‑based front end with offset trimming to saturation, run PV on‑device, and expose minute‑level RR with a compact signal‑quality index. This arrangement balances accuracy, robustness, and power.

A natural extension of this blueprint is sensor fusion, which becomes especially valuable outside the lab. Because chest motion is spatially heterogeneous and local artifacts are common (e.g., a strap shift on one side), fusing multiple strain channels—possibly with different geometries and orientations—can suppress outliers and stabilize RR estimates. Practical fusion can be lightweight: a quality‑weighted median of the per‑minute RR from left‑vertical, right‑vertical, and a circular midline channel, where weights come from simple signal‑quality indicators (cycle amplitude stability, inter‑cycle variability, and fraction of valid cycles detected), already offers robustness without heavy computation. A step up in sophistication is a one‑dimensional Kalman filter that treats RR as a slowly varying state and uses each sensor as a measurement with noise set by its quality score; this yields smooth estimates and principled confidence intervals while remaining MCU‑friendly. Cross‑sensor gating—temporarily ignoring a channel when its quality drops—or majority‑vote logic during disagreements further protects against transient artifacts. Finally, incorporating an auxiliary modality such as an IMU for posture detection can inform the fusion (e.g., reweight lateral sensors when the user lies supine).

There are practical considerations for translation. Comfort and durability must coexist with signal quality: encapsulation should manage moisture without trapping heat, interconnects should follow low‑strain garment seams, and closures should deliver repeatable tension without over‑compression. Washability, sweat ingress, and don/doff repeatability are likely failure modes and should be engineered and tested with the same rigor as the electronics and algorithms. From an application perspective, minute‑level RR is often sufficient for wellness and early‑warning use cases, but clinical decision support will also benefit from confidence metrics, artifact flags, and explicit handling of speech and ambulation.

We emphasize that the following design implications should be interpreted as preliminary, given the pilot scope and the controlled quiet-breathing protocol (no paced shallow/deep/irregular breathing trials were included). This is a pilot with a small cohort and minute‑level metrics against a thermistor reference; free‑living conditions, breath‑by‑breath agreement, and long‑term wear were outside scope. In addition, our primary accuracy summaries are computed at the 1‑min window level and then pooled across participants. While appropriate for descriptive evaluation in a pilot, this pooling increases the apparent sample size and does not independently quantify inter-subject variability. Future studies will report subject-level metrics and use hierarchical/repeated-measures analyses to separate within-subject and between-subject variability. The left–right asymmetry we observed may reflect body habitus and belt fit and should not be over‑generalized. Future work will therefore expand to larger, more diverse cohorts; compare against clinical gold standards (spirometry or pneumotachography) using Bland–Altman, correlation, and error‑distribution analyses; characterize performance across postures and activities in free‑living studies; and quantify durability and wash cycles of the printed stack. On the algorithmic side we will implement the fusion strategies outlined above, define and validate real‑time signal‑quality indices, evaluate motion‑aware reweighting using IMU cues, and explore adaptive belt‑tension compensation. System integration with flexible batteries/supercapacitors and on‑device analytics will enable fully self‑contained garments.

In conclusion, a printed e‑textile belt can provide minute-level RR estimates with reasonable agreement during quiet breathing in this pilot study, when sensor orientation and placement are chosen to respect thoracic biomechanics and when estimation methods acknowledge the non‑stationary nature of breathing. In our data, vertical serpentines on the lateral thorax were the most reliable configuration and a lightweight PV detector outperformed PSD. These pilot observations suggest preliminary design guidance for robust, low-power RR wearables, and motivate validation in broader conditions.

Beyond its utility as a vital sign, respiration monitoring carries broader clinical significance: respiratory rhythm and variability are tightly coupled to autonomic nervous system function. Continuous, unobtrusive tracking of breathing patterns can therefore serve not only to detect respiratory compromise in patients but also as a surrogate marker of autonomic balance, complementing heart rate variability and other physiological indices. Such integration opens avenues for monitoring stress, sleep quality, and recovery in everyday life, while in clinical contexts it may provide early warning of dysautonomia, cardiopulmonary instability, or impaired vagal tone. By embedding reliable respiration sensing into garments, we enable longitudinal assessment of both pulmonary health and autonomic regulation, extending the impact of wearable systems from simple rate detection to richer insights into systemic physiology.

## Supplementary Information

Below is the link to the electronic supplementary material.

Supplementary Material 1.

## Data Availability

All data supporting the reported results are contained within this article. Additional data and code can be made available on request from the corresponding authors.
