# Supplementary material for: Effects of sensor geometry, placement, and cycle detection on wearable respiration monitoring with textile printed strain sensors
Source: Discov Sens. 2026 Mar 27;2(1):22. doi: 10.1007/s44397-026-00054-0 (PMC13021712; doi:10.1007/s44397-026-00054-0)
Supplement: Supplementary file 1 — Supplementary Material 1. [file 44397_2026_54_MOESM1_ESM.pdf]

Support Information File

Effects of sensor geometry, placement, and cycle detection on wearable respiration monitoring with textile printed strain sensors

Manuel Reis Carneiro[1,2*], João Silva[2], Mahmoud Tavakoli[2*]

1 - Department of Health Sciences and Technology, ETH Zürich, Zürich 8008, Switzerland; manuel.reiscarneiro@hest.ethz.ch

2 - Institute of Systems and Robotics, Department of Electrical and Computer Engineering, University of Coimbra, Coimbra 3030-290, Portugal; mahmoud@isr.uc.pt

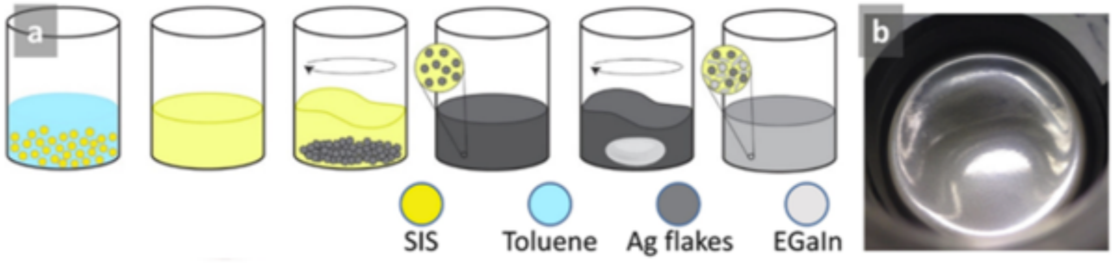

Figure S1: (a) Representative sequence of steps to make the conductive ink; (b) The result ink has coherent and shiny appearance

a

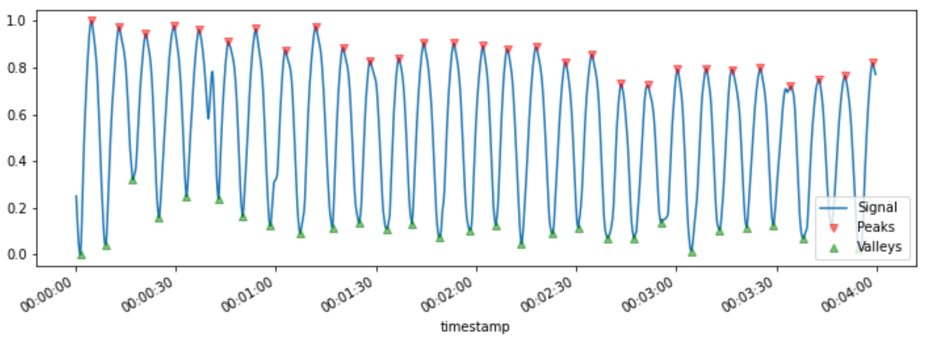

b

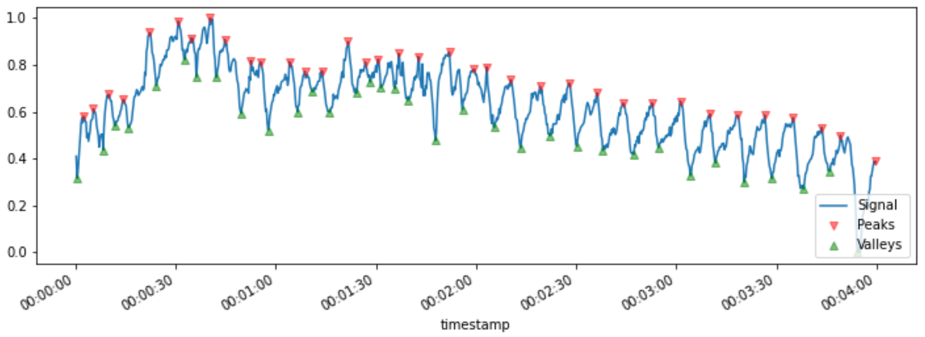

Figure S2: Demonstration of the PV algorithm with the peaks and valleys that the algorithm found. a) PV for the 4 minute test of Thermistor for subject 1 b) PV for the 4 minute test of the Circular sensor on the left side of the ribs for subject 1.

a

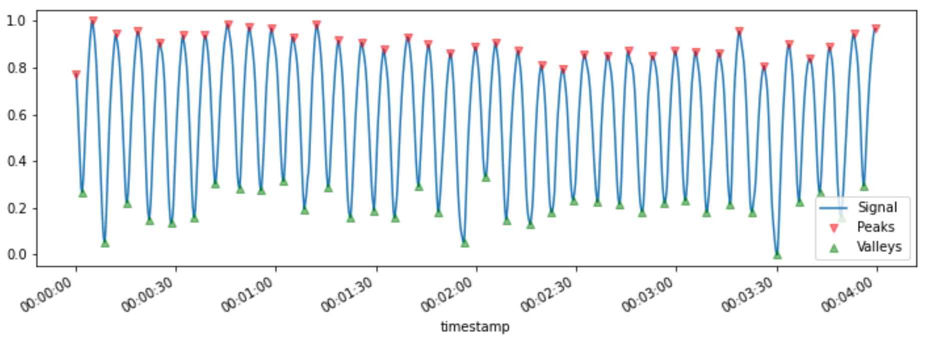

b

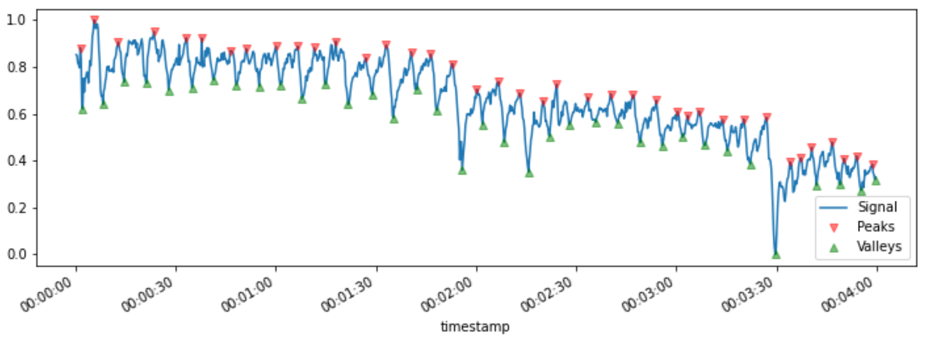

Figure S3: Demonstration of the PV algorithm with the peaks and valleys that the algorithm found. (a) PV for the 4 minute test of Thermistor for subject 2 (b) PV for the 4 minute test of the Circular sensor on the left side of the ribs for subject 2.

a

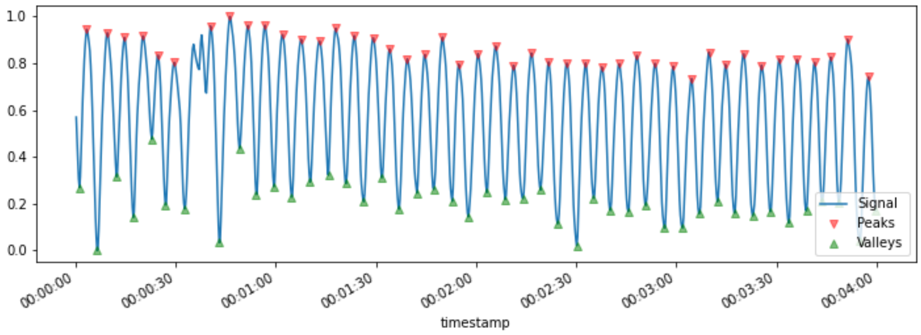

b

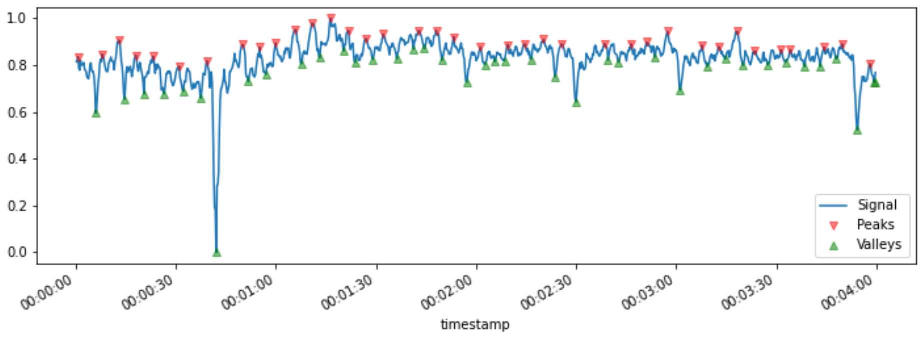

Figure S4: Demonstration of the PV algorithm with the peaks and valleys that the algorithm found. (a) PV for the 4 minute test of Thermistor for subject 3 (b) PV for the 4 minute test of the Circular sensor on the left side of the ribs for subject 3.

a

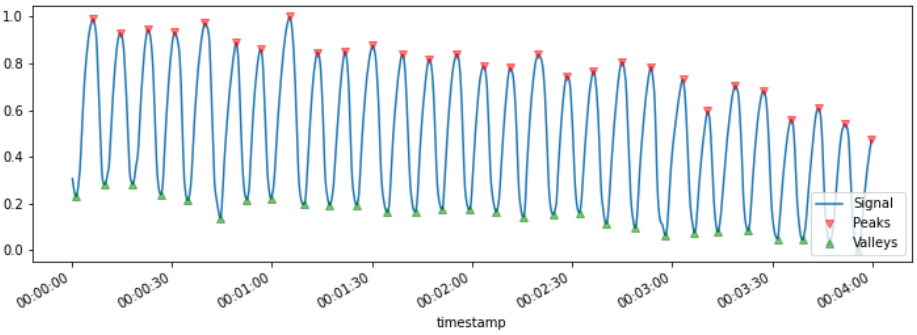

b

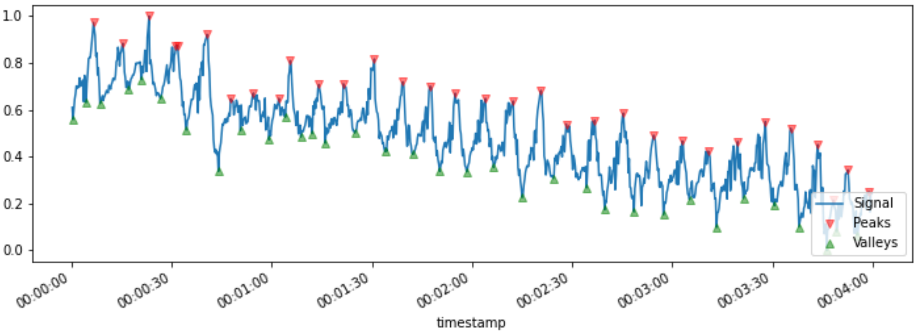

Figure S5: Demonstration of the PV algorithm with the peaks and valleys that the algorithm found. (a) PV for the 4 minute test of Thermistor for subject 1 (b) PV for the 4 minute test of the Circular sensor on the center of the chest for subject 1.

a

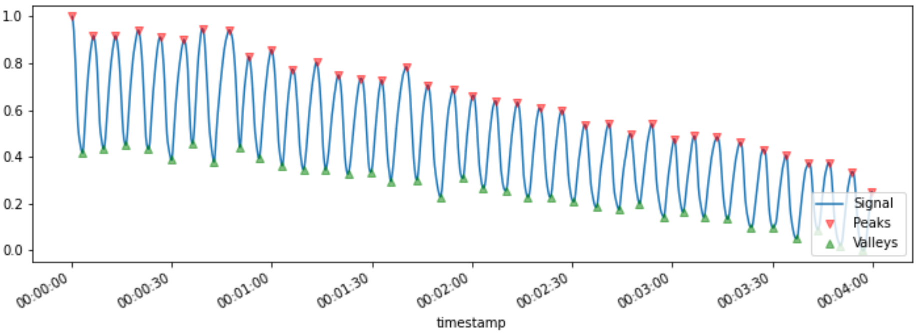

b

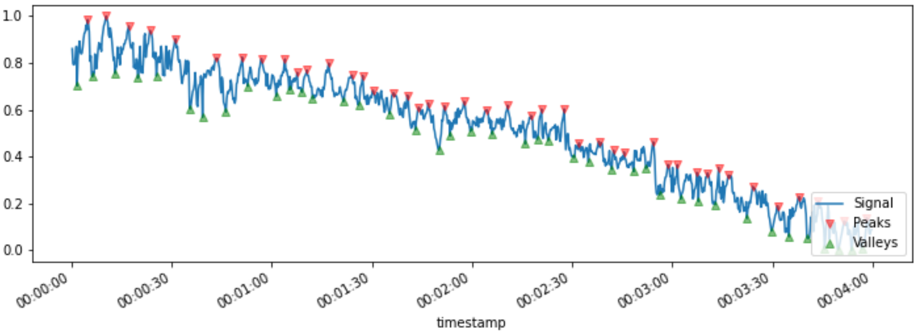

Figure S6: Demonstration of the PV algorithm with the peaks and valleys that the algorithm found. (a) PV for the 4 minute test of Thermistor for subject 2 (b) PV for the 4 minute test of the Circular sensor on the center of the chest for subject 2.

a

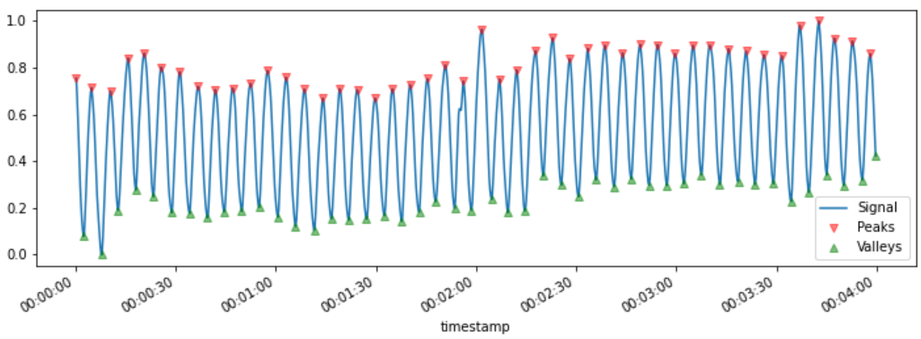

b

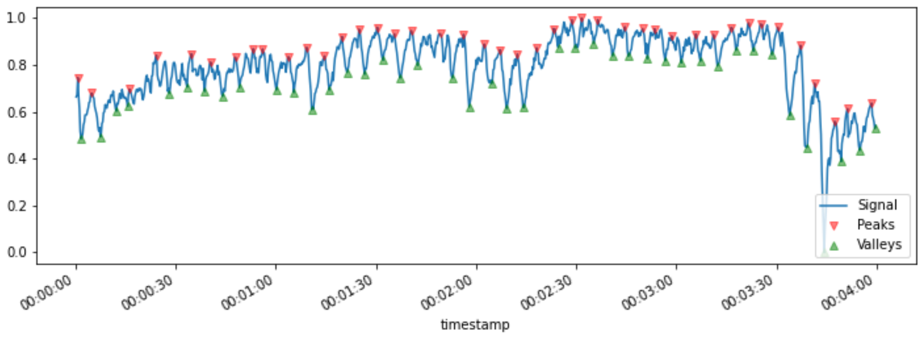

Figure S7: Demonstration of the PV algorithm with the peaks and valleys that the algorithm found. (a) PV for the 4 minute test of Thermistor for subject 3 (b) PV for the 4 minute test of the Circular sensor on the center of the chest for subject 3

a

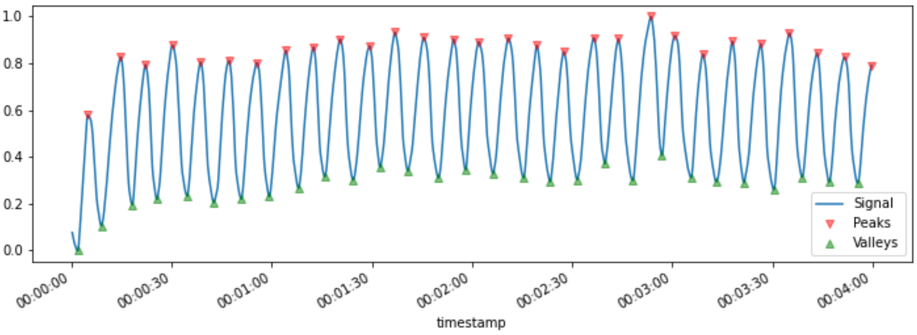

b

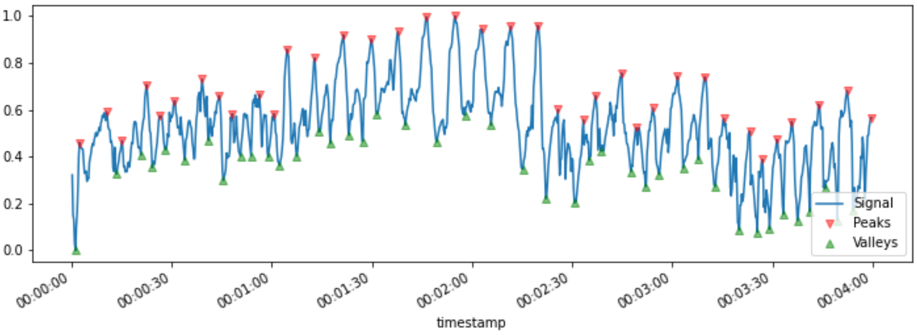

Figure S8: Demonstration of the PV algorithm with the peaks and valleys that the algorithm found. (a) PV for the 4 minute test of Thermistor for subject 1 (b) PV for the 4 minute test of the Circular sensor on the right side of the ribs for subject 1

a

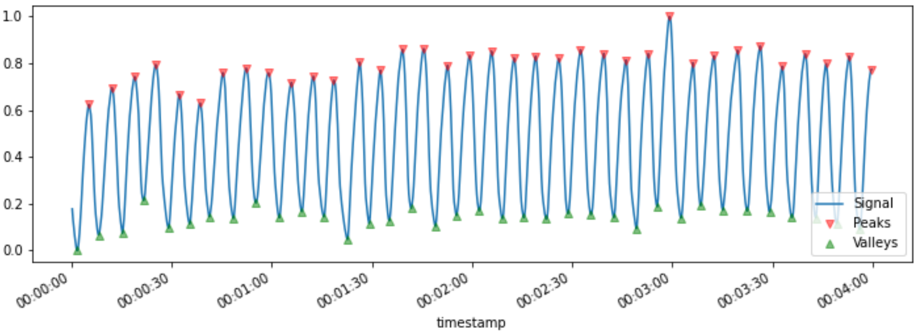

b

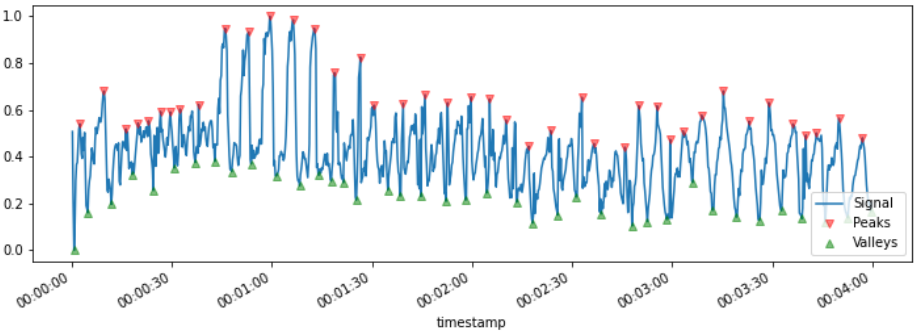

Figure S9: Demonstration of the PV algorithm with the peaks and valleys that the algorithm found. (a) PV for the 4 minute test of Thermistor for subject 2 (b) PV for the 4 minute test of the Circular sensor on the right side of the ribs for subject 2.

a

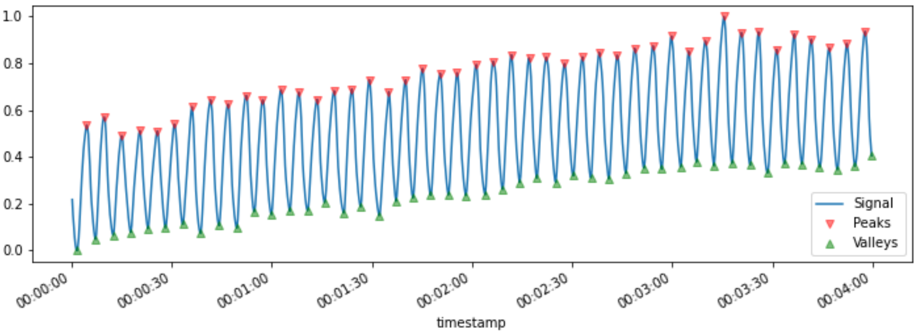

b

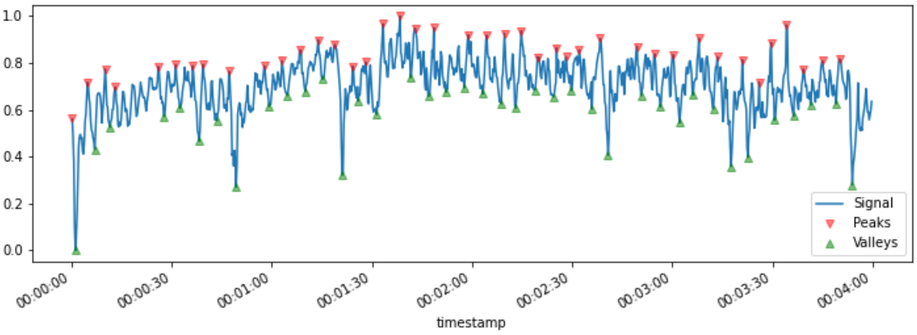

Figure S10: Demonstration of the PV algorithm with the peaks and valleys that the algorithm found. (a) PV for the 4 minute test of Thermistor for subject 3 (b) PV for the 4 minute test of the Circular sensor on the right side of the ribs for subject 3.

a

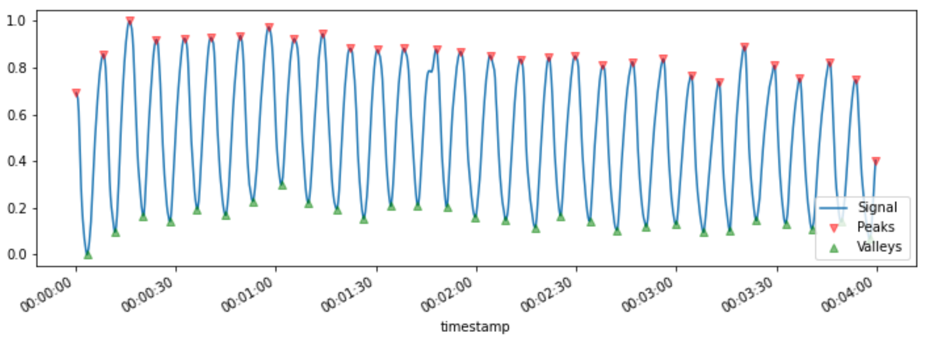

b

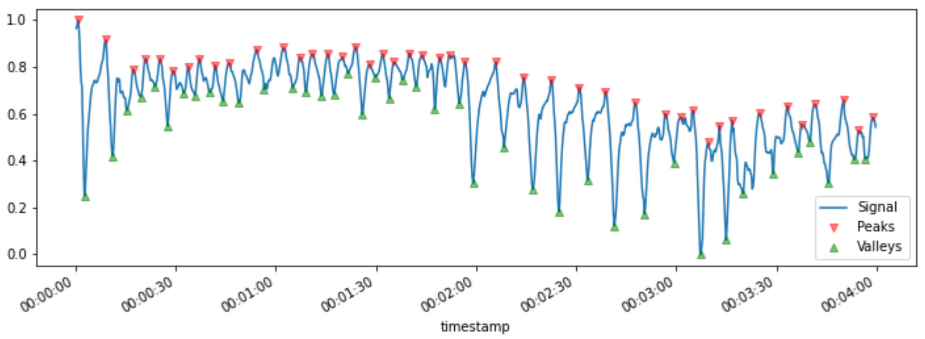

Figure S11: Demonstration of the PV algorithm with the peaks and valleys that the algorithm found. (a) PV for the 4 minute test of Thermistor for subject 1 (b) PV for the 4 minute test of the Linear sensor with horizontal orientation on the left side of the ribs for subject 1.

a

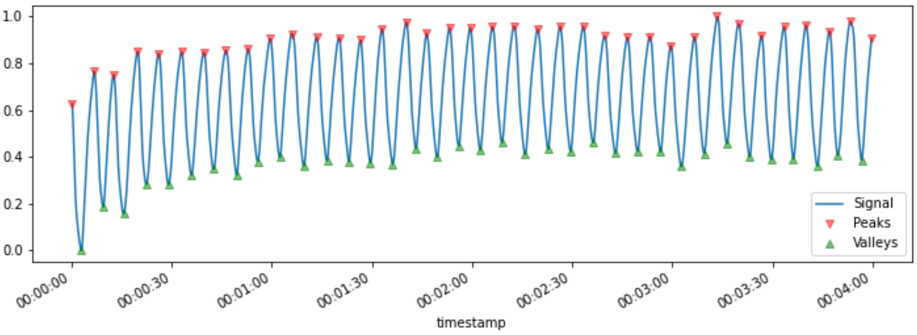

b

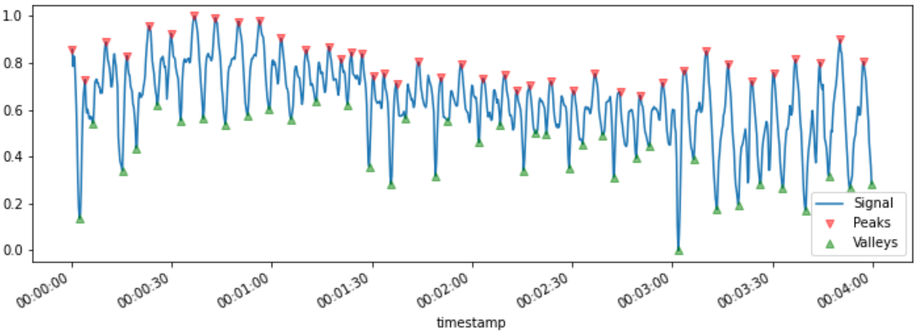

Figure S12: Demonstration of the PV algorithm with the peaks and valleys that the algorithm found. (a) PV for the 4 minute test of Thermistor for subject 2 (b) PV for the 4 minute test of the Linear sensor with horizontal orientation on the left side of the ribs for subject 2.

a

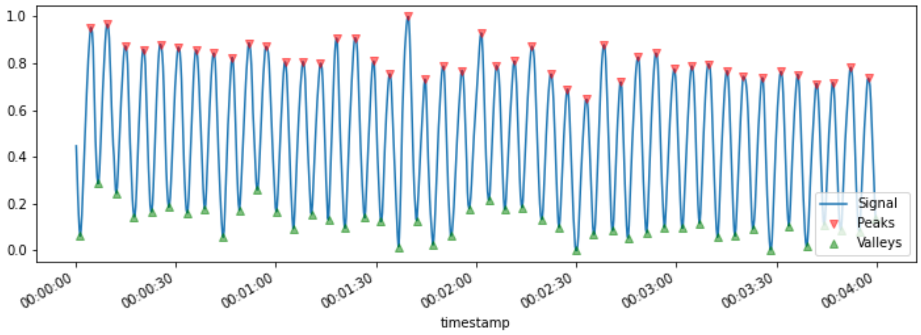

b

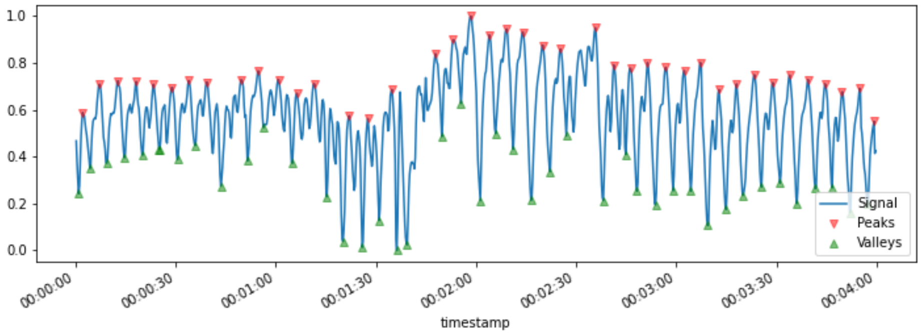

Figure S13: Demonstration of the PV algorithm with the peaks and valleys that the algorithm found. (a) PV for the 4 minute test of Thermistor for subject 3 (b) PV for the 4 minute test of the Linear sensor with horizontal orientation on the left side of the ribs forsubject 3

a

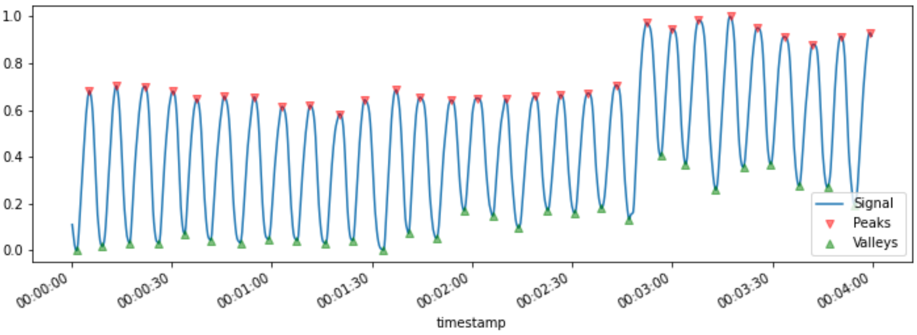

b

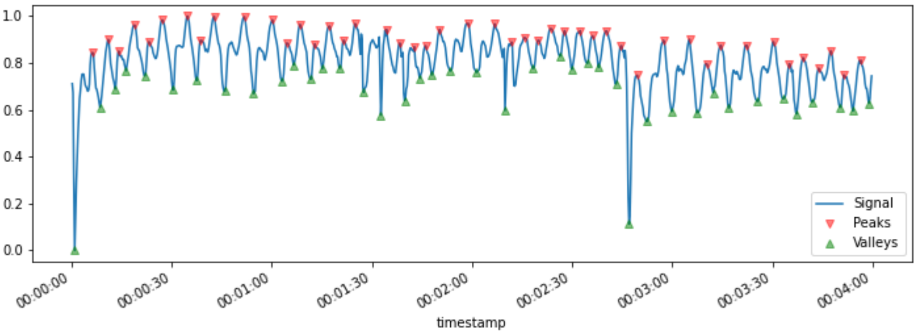

Figure S14: Demonstration of the PV algorithm with the peaks and valleys that the algorithm found. (a) PV for the 4 minute test of Thermistor for subject 1 (b) PV for the 4 minute test of the Linear sensor with horizontal orientation on the center of the chest for subject 1.

a

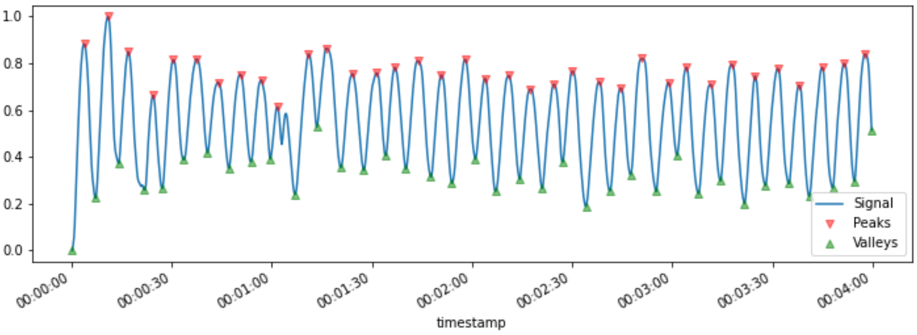

b

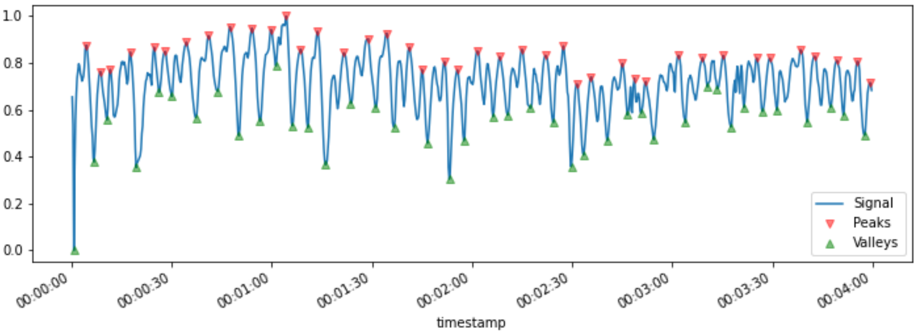

Figure S15: Demonstration of the PV algorithm with the peaks and valleys that the algorithm found. (a) PV for the 4 minute test of Thermistor for subject 2 (b) PV for the 4 minute test of the Linear sensor with horizontal orientation on the center of the chest for subject 2.

a

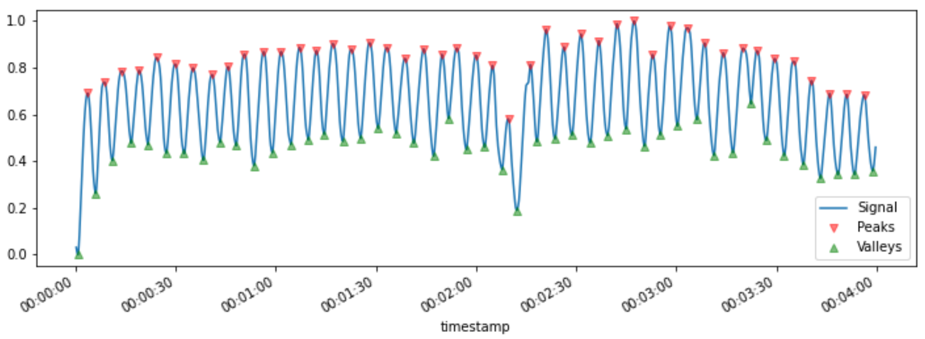

b

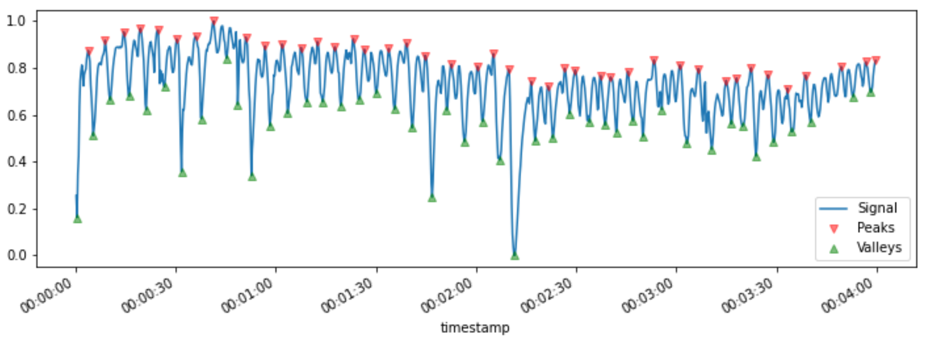

Figure S16: Demonstration of the PV algorithm with the peaks and valleys that the algorithm found. (a) PV for the 4 minute test of Thermistor for subject 3 (b) PV for the 4 minute test of the Linear sensor with horizontal orientation on the center of the chest for subject 3.

a

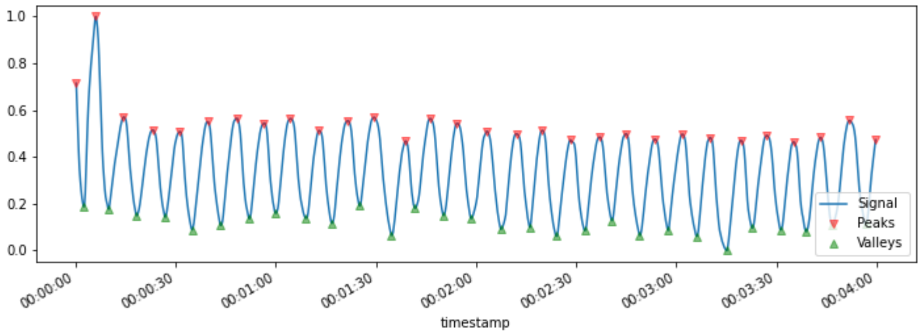

b

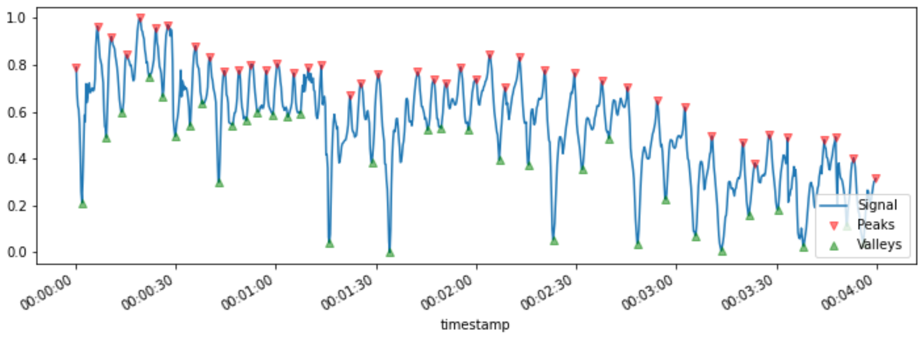

Figure S17: Demonstration of the PV algorithm with the peaks and valleys that the algorithm found. (a) PV for the 4 minute test of Thermistor for subject 1 (b) PV for the 4 minute test of the Linear sensor with horizontal orientation on the right side of the ribs for subject 1.

a

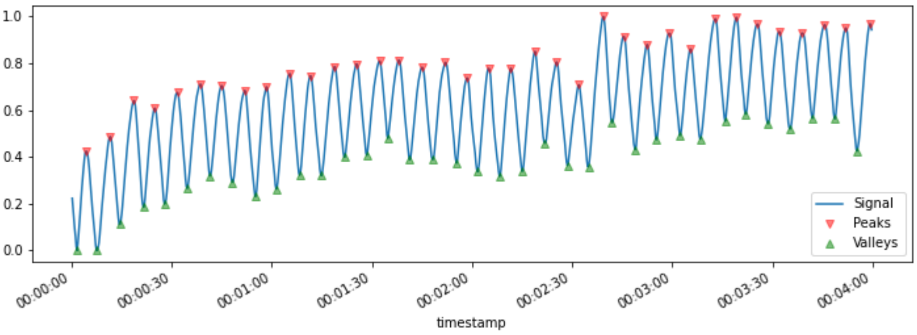

b

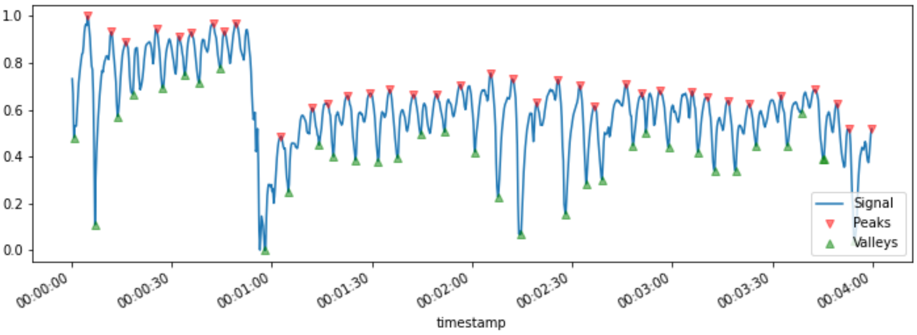

Figure S18: Demonstration of the PV algorithm with the peaks and valleys that the algorithm found. (a) PV for the 4 minute test of Thermistor for subject 2 (b) PV for the 4 minute test of the Linear sensor with horizontal orientation on the right side of the ribs for subject 2.

a

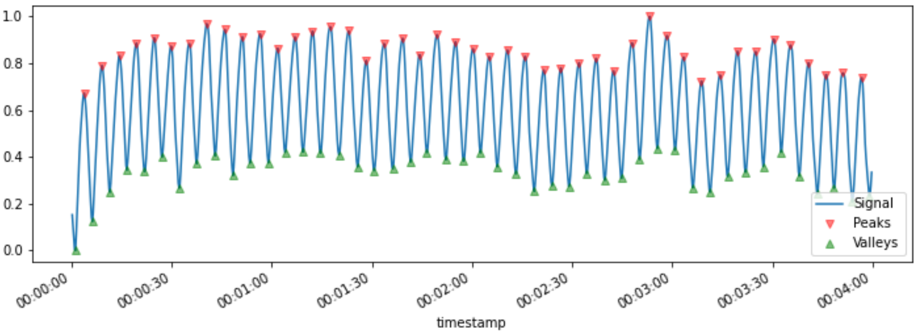

b

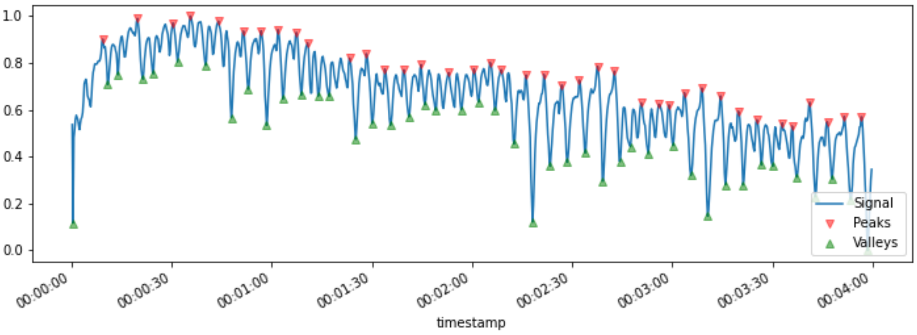

Figure S19: Demonstration of the PV algorithm with the peaks and valleys that the algorithm found. (a) PV for the 4 minute test of Thermistor for subject 3 (b) PV for the 4 minute test of the Linear sensor with horizontal orientation on the right side of the ribs for subject 3.

a

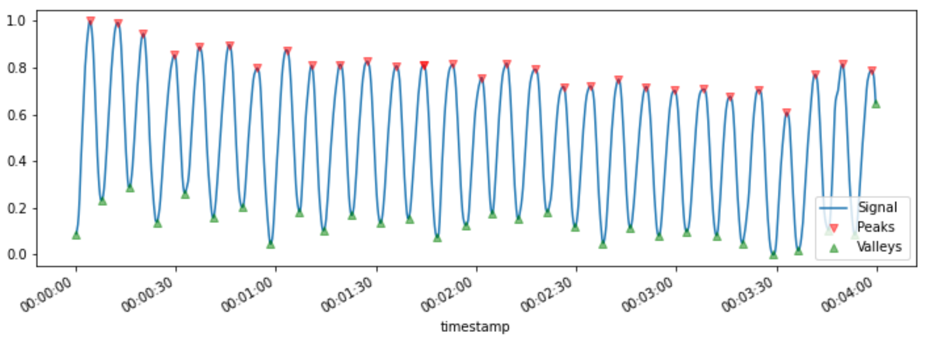

b

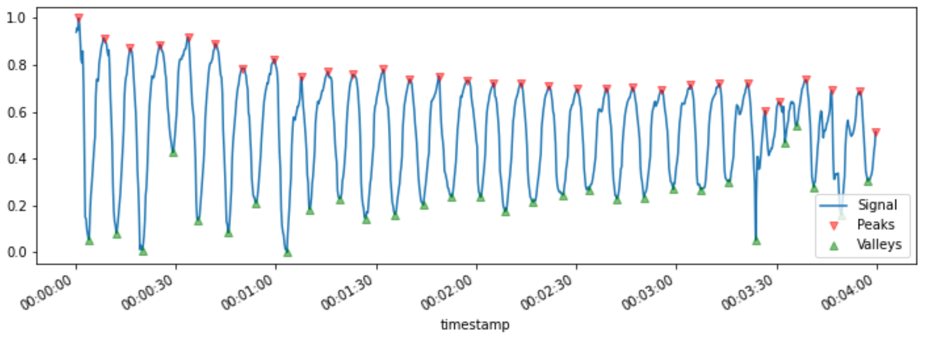

Figure S20: Demonstration of the PV algorithm with the peaks and valleys that the algorithm found. (a) PV for the 4 minute test of Thermistor for subject 2 (b) PV for the 4 minute test of the Linear sensor with vertical orientation on the left side of the ribs for subject 2.

a

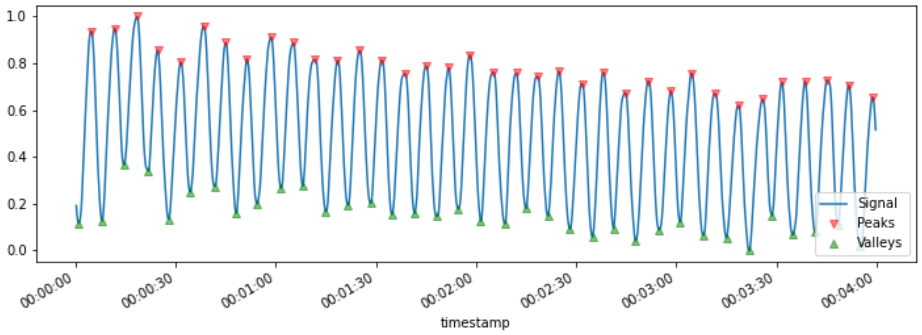

b

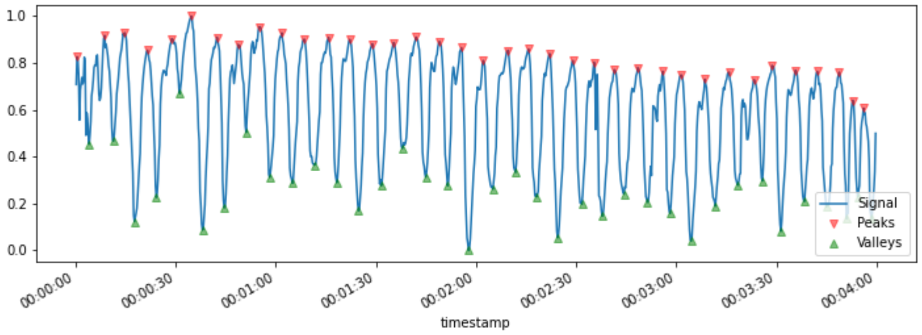

Figure S21: Demonstration of the PV algorithm with the peaks and valleys that the algorithm found. (a) PV for the 4 minute test of Thermistor for subject 2 (b) PV for the 4 minute test of the Linear sensor with vertical orientation on the left side of the ribs for subject 2

a

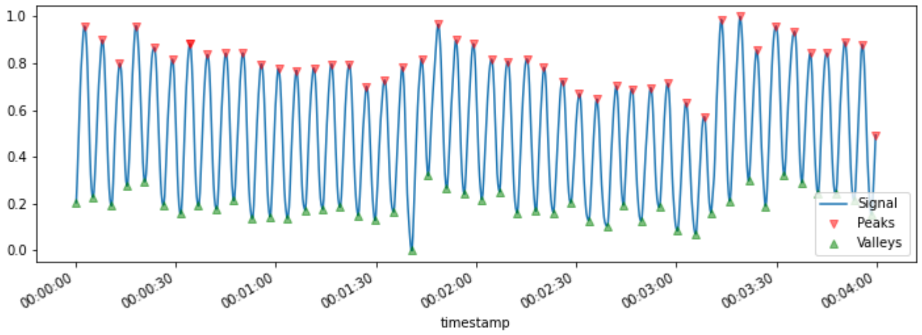

b

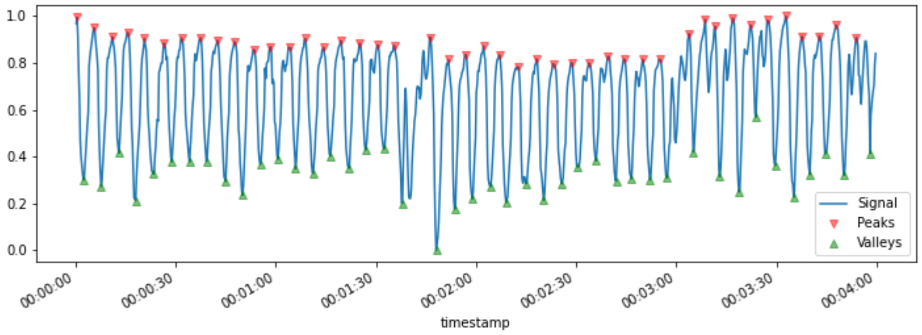

Figure S22: Demonstration of the PV algorithm with the peaks and valleys that the algorithm found. (a) PV for the 4 minute test of Thermistor for subject 3 (b) PV for the 4 minute test of the Linear sensor with vertical orientation on the left side of the ribs for subject 3.

a

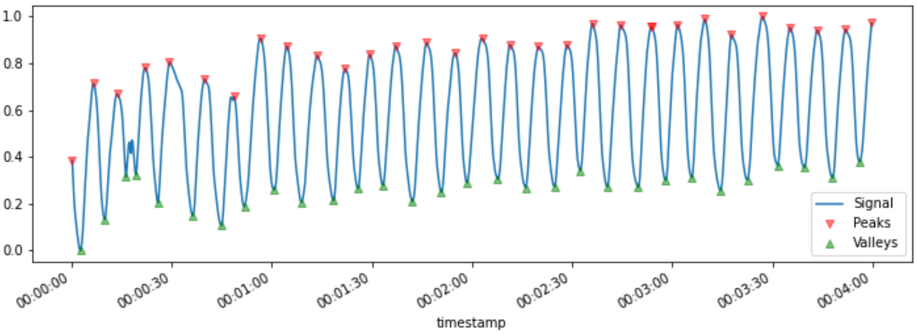

b

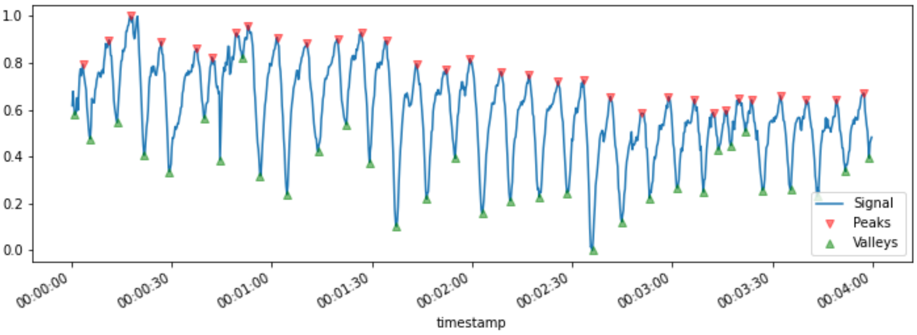

Figure S23: Demonstration of the PV algorithm with the peaks and valleys that the algorithm found. (a) PV for the 4 minute test of Thermistor for subject 1 (b) PV for the 4 minute test of the Linear sensor with vertical orientation on the center of the chest for subject 1

a

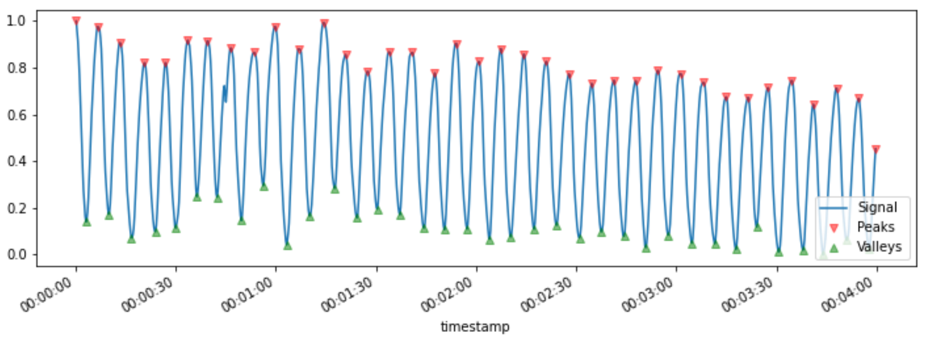

b

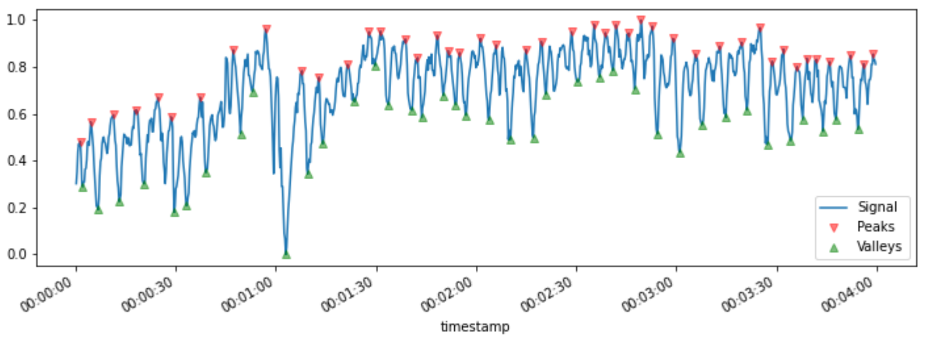

Figure S24: Demonstration of the PV algorithm with the peaks and valleys that the algorithm found. (a) PV for the 4 minute test of Thermistor for subject 2 (b) PV for the 4 minute test of the Linear sensor with vertical orientation on the center of the chest for subject 2

a

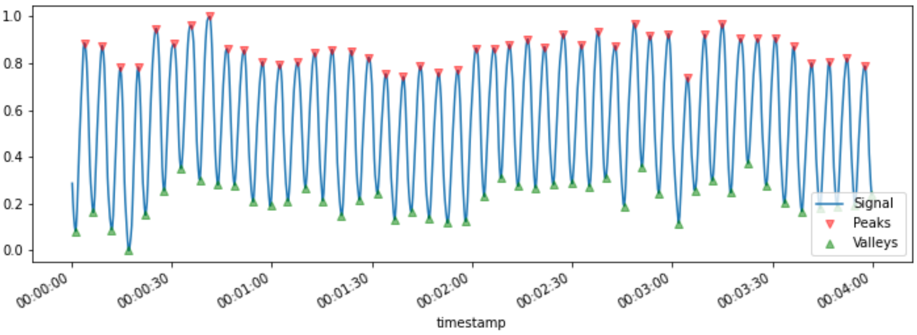

b

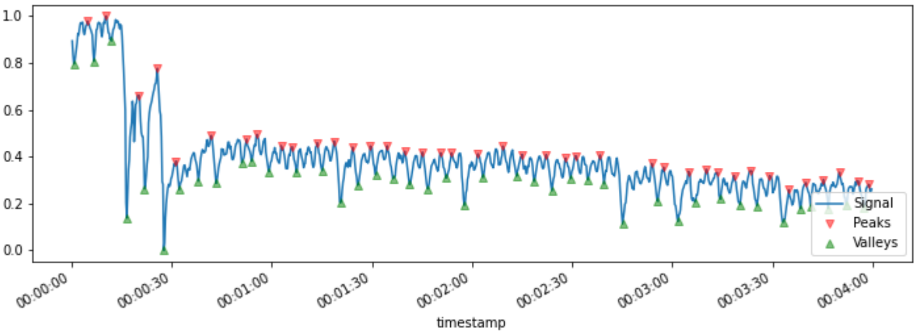

Figure S25: Demonstration of the PV algorithm with the peaks and valleys that the algorithm found. (a) PV for the 4 minute test of Thermistor for subject 3 (b) PV for the 4 minute test of the Linear sensor with vertical orientation on the center of the chest for subject 3

a

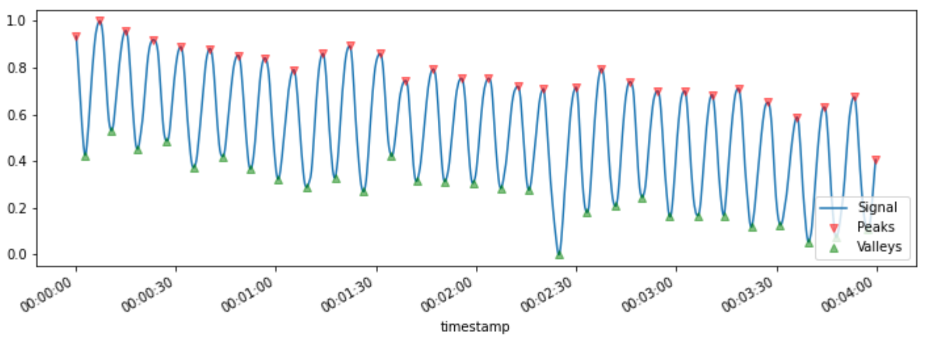

b

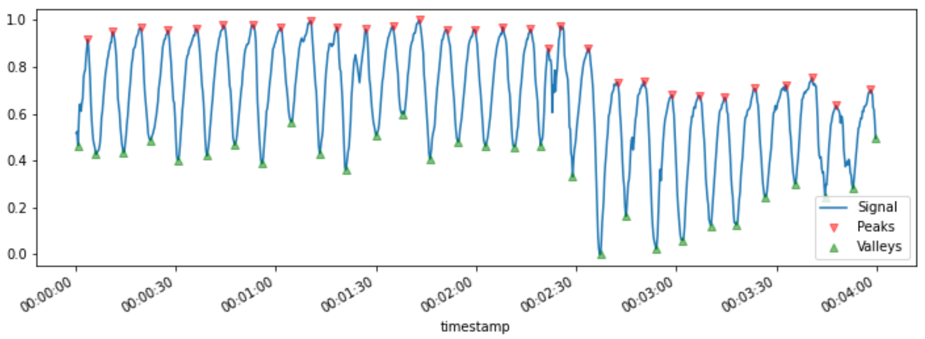

Figure S26: Demonstration of the PV algorithm with the peaks and valleys that the algorithm found. (a) PV for the 4 minute test of Thermistor for subject 1 (b) PV for the 4 minute test of the Linear sensor with vertical orientation on the right side of the ribs for subject 1

a

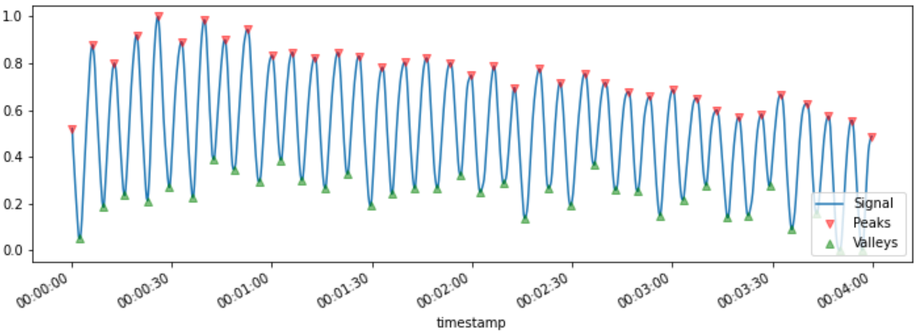

b

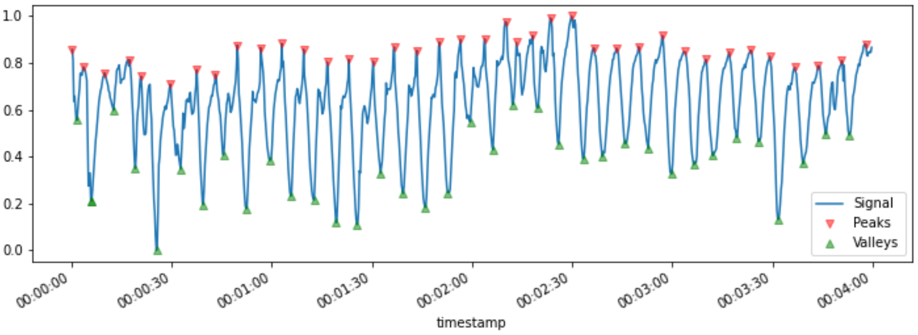

Figure S27: Demonstration of the PV algorithm with the peaks and valleys that the algorithm found. (a) PV for the 4 minute test of Thermistor for subject 2 (b) PV for the 4 minute test of the Linear sensor with vertical orientation on the right side of the ribs for subject 2

a

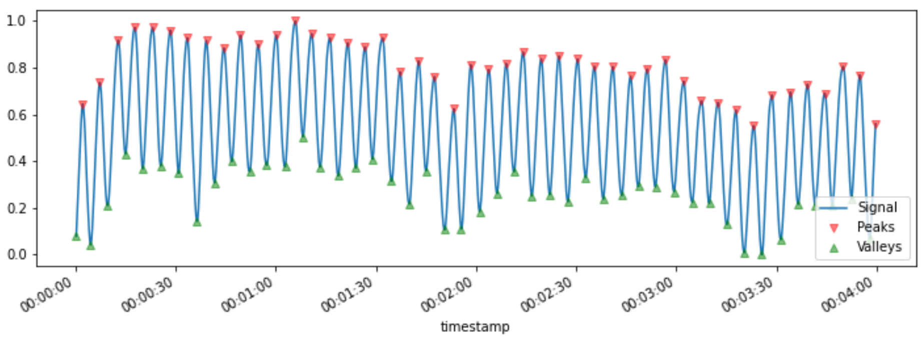

b

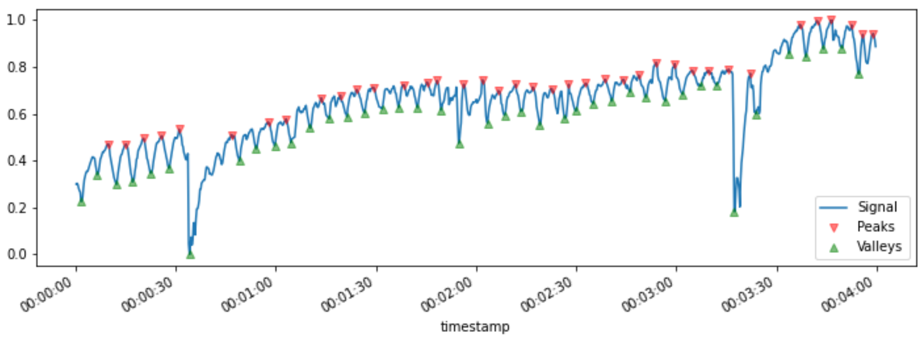

Figure S28: Demonstration of the PV algorithm with the peaks and valleys that the algorithm found. (a) PV for the 4 minute test of Thermistor for subject 3 (b) PV for the 4 minute test of the Linear sensor with vertical orientation on the right side of the ribs for subject 3.

Table S1: Detailed view on the Circular sensor for the 4 minute test with the PV algorythm.

Figure	Subject	Sensor Position	Thermistor Measure	Sensor Measure	Error
4.1	1	Circular on Left	7,4	10,44	3,04
A.1	2	Circular on Left	9,24	9,56	0,32
A.2	3	Circular on Left	10,3	9,12	1,18
A.3	1	Circular on Center	7,18	8,18	1,00
A.4	2	Circular on Center	9,05	8,35	0,70
A.5	3	Circular on Center	12,04	9,27	2,77
A.6	1	Circular on Right	8,04	10,3	2,26
A.7	2	Circular on Right	9,52	10,53	1,01
A.8	3	Circular on Right	11,33	9,39	1,94

Table S2: Detailed view on the Linear sensor with the Horizontal Orientation for the 4minute test with the PV algorythm.

Figure	Subject	Sensor Position	Thermistor Measure	Sensor Measure	Error
A.9	1	Linear on Left	7,79	11,75	3,96
A.10	2	Linear on Left	9,13	10,18	1,05
A.11	3	Linear on Left	11,28	11,92	0,64
A.12	1	Linear on Center	7,31	10,08	2,77
A.13	2	Linear on Center	9,09	10,81	1,72
A.14	3	Linear on Center	11,33	10,39	0,94
A.15	1	Linear on Right	8,00	13,16	5,16
A.16	2	Linear on Right	9,28	9,94	0,66
A.17	3	Linear on Right	11,42	10,50	0,92

Table S3: Detailed view on the Linear sensor with the Vertical Orientation for the 4 minute test with the PV algorythm.

**Figure**	**Subject**	**Sensor Position**	**Thermistor Measure**	**Sensor Measure**	**Error**
A.18	1	Linear on Left	7,73	7,18	0,55
A.19	2	Linear on Left	9,17	9,09	0,08
A.20	3	Linear on Left	12,57	11,89	0,68
A.21	1	Linear on Center	8,38	9,18	0,80
A.22	2	Linear on Center	9,05	9,44	0,39
A.23	3	Linear on Center	11,28	8,20	3,08
A.24	1	Linear on Right	7,96	8,18	0,22
A.25	2	Linear on Right	9,05	10,40	1,35
A.26	3	Linear on Right	12,09	7,24	

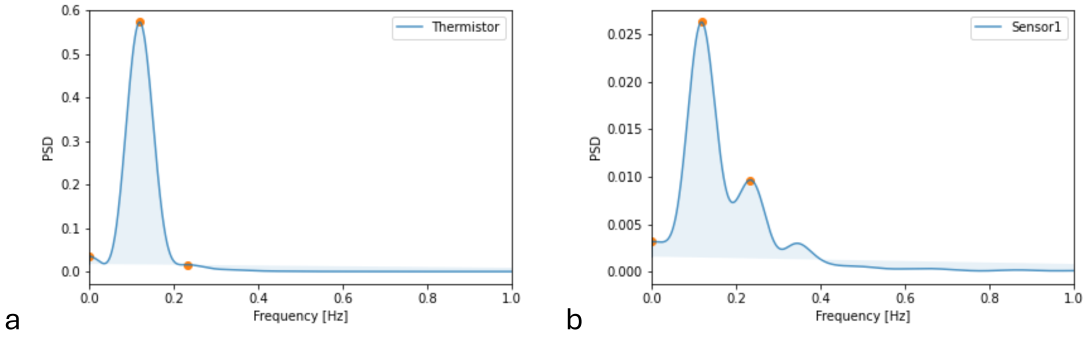

Figure S29: Demonstration of the PSD graphic where it shows the 3 most important frequencies of the singal. a) PSD for the 4 minute test of Thermistor for subject 1 b) PSD for the 4 minute test of the Circular sensor on the left side of the ribs for subject 1.

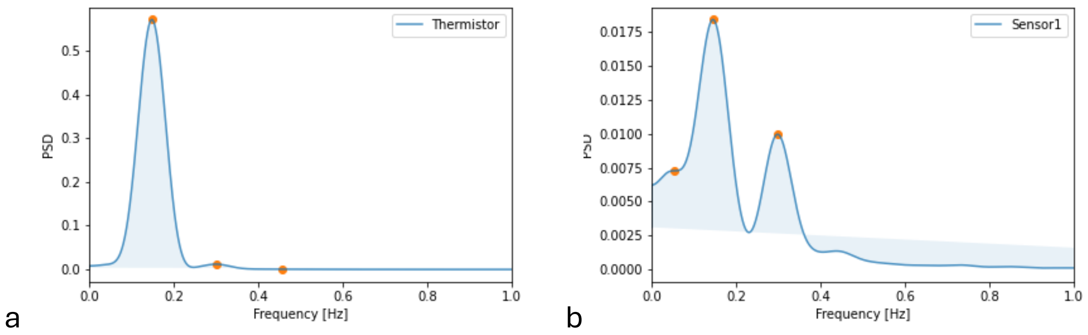

Figure S30: Demonstration of the PSD algorithm with the peaks and valleys that the algorithm found.(a) PSD for the 4 minute test of Thermistor for subject 2 (b) PSD for the 4 minute test of the Circular sensor on the left side of the ribs for subject 2.

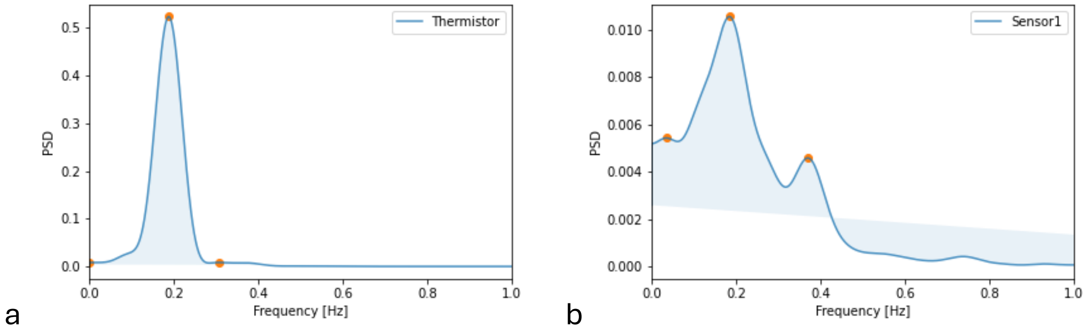

Figure S31: Demonstration of the PSD algorithm with the peaks and valleys that the algorithm found.(a) PSD for the 4 minute test of Thermistor for subject 3 (b) PSD for the 4 minute test of the Circular sensor on the left side of the ribs for subject 3.

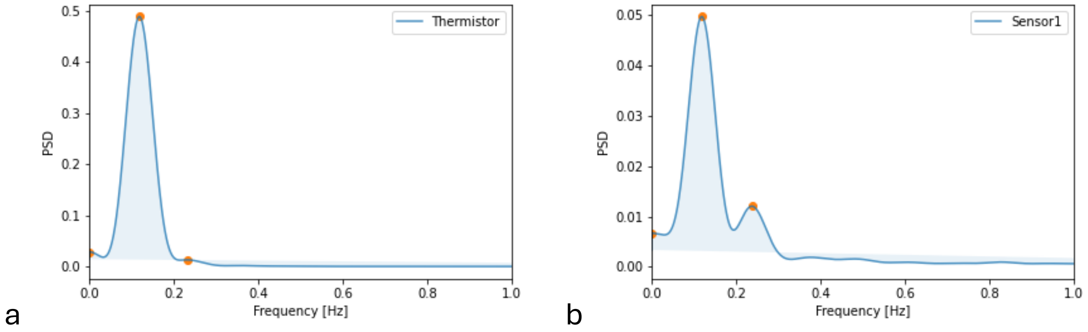

Figure S32: Demonstration of the PSD algorithm with the peaks and valleys that the algorithm found.(a) PSD for the 4 minute test of Thermistor for subject 1 (b) PSD for the 4 minute test of the Circular sensor on the center of the chest for subject 1.

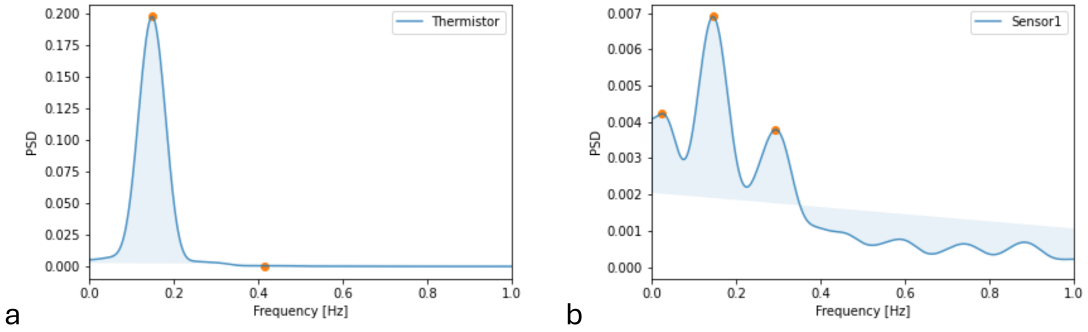

Figure S33: Demonstration of the PSD algorithm with the peaks and valleys that the algorithm found.(a) PSD for the 4 minute test of Thermistor for subject 2 (b) PSD for the 4 minute test of the Circular sensor on the center of the chest for subject 2.

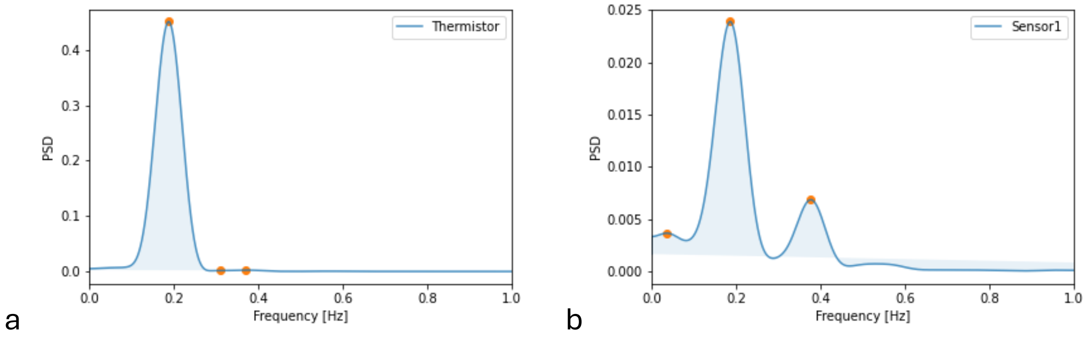

Figure S34: Demonstration of the PSD algorithm with the peaks and valleys that the algorithm found.(a) PSD for the 4 minute test of Thermistor for subject 3 (b) PSD for the 4 minute test of the Circular sensor on the center of the chest for subject 3.

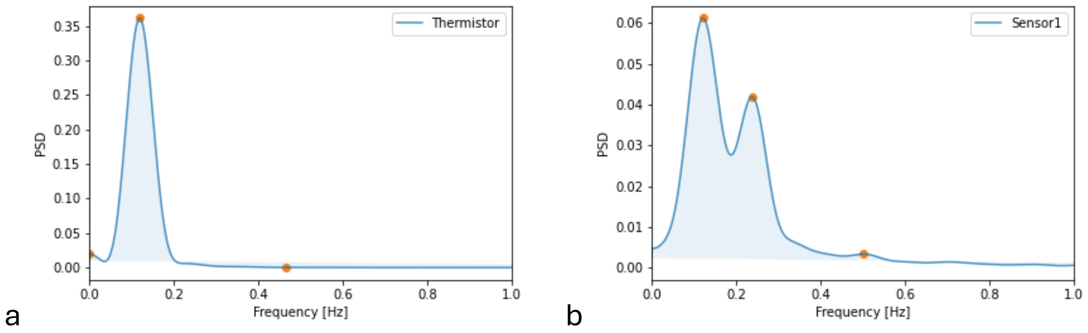

Figure S35: Demonstration of the PSD algorithm with the peaks and valleys that the algorithm found.(a) PSD for the 4 minute test of Thermistor for subject 1 (b) PSD for the 4 minute test of the Circular sensor on the right side of the ribs for subject 1.

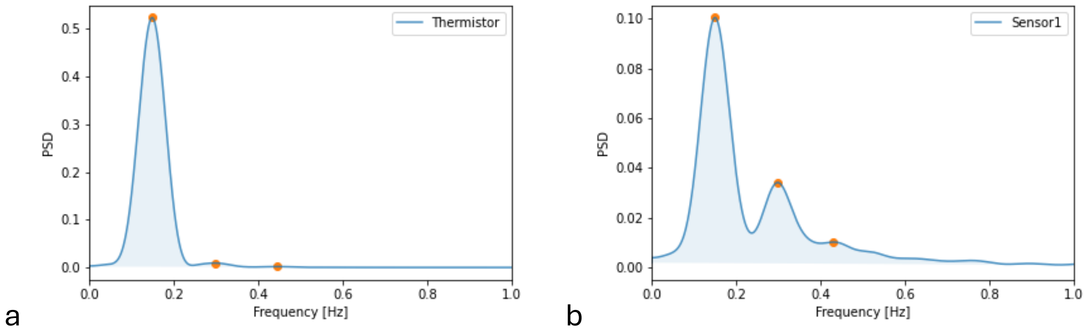

Figure S36: Demonstration of the PSD algorithm with the peaks and valleys that the algorithm found.(a) PSD for the 4 minute test of Thermistor for subject 2 (b) PSD for the 4 minute test of the Circular sensor on the right side of the ribs for subject 2.

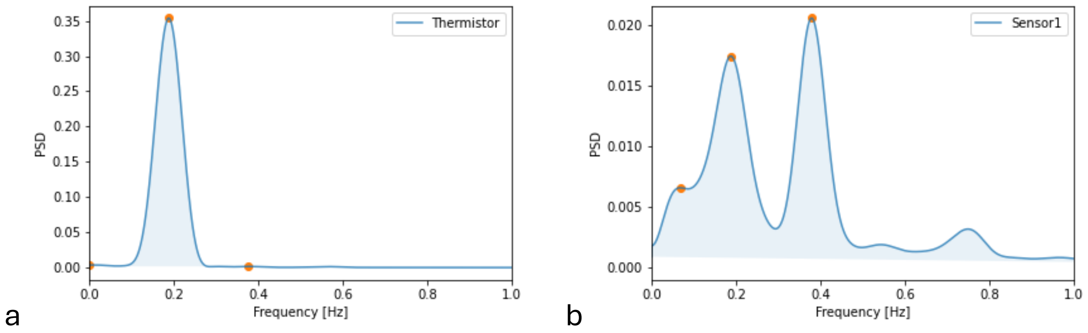

Figure S37: Demonstration of the PSD algorithm with the peaks and valleys that the algorithm found.(a) PSD for the 4 minute test of Thermistor for subject 3 (b) PSD for the 4 minute test of the Circular sensor on the right side of the ribs for subject 3.

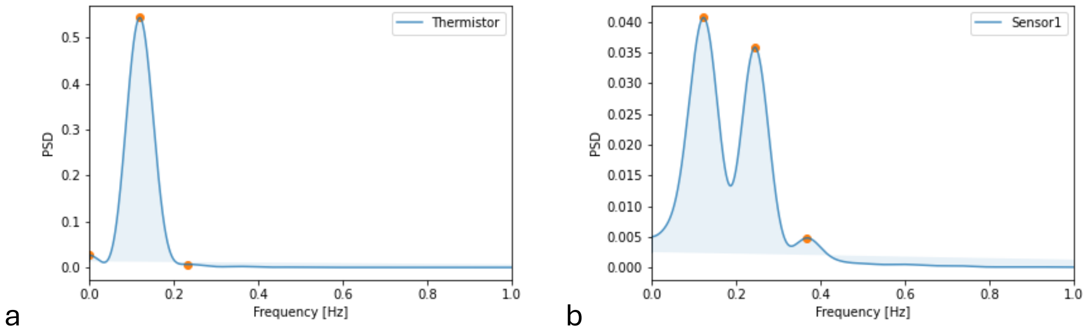

Figure S38: Demonstration of the PSD algorithm with the peaks and valleys that the algorithm found.(a) PSD for the 4 minute test of Thermistor for subject 1 (b) PSD for the 4 minute test of the Linear sensor with horizontal orientation on the left side of the ribs for subject 1.

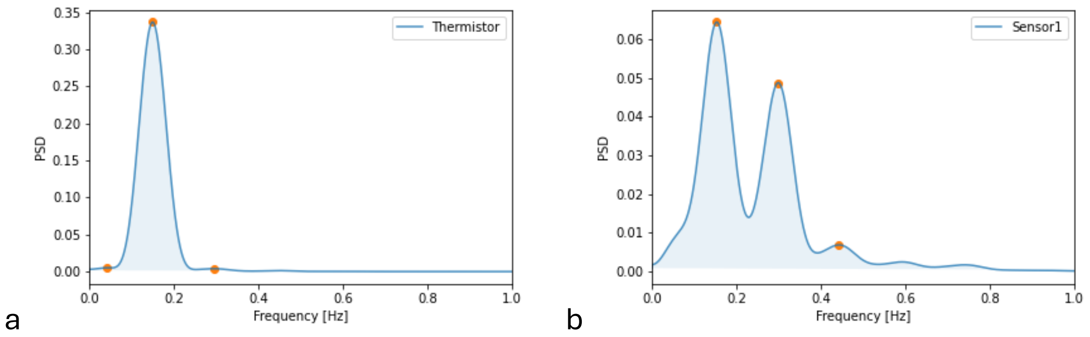

Figure S39: Demonstration of the PSD algorithm with the peaks and valleys that the algorithm found.(a) PSD for the 4 minute test of Thermistor for subject 2 (b) PSD for the 4 minute test of the Linear sensor with horizontal orientation on the left side of the ribs for subject 2.

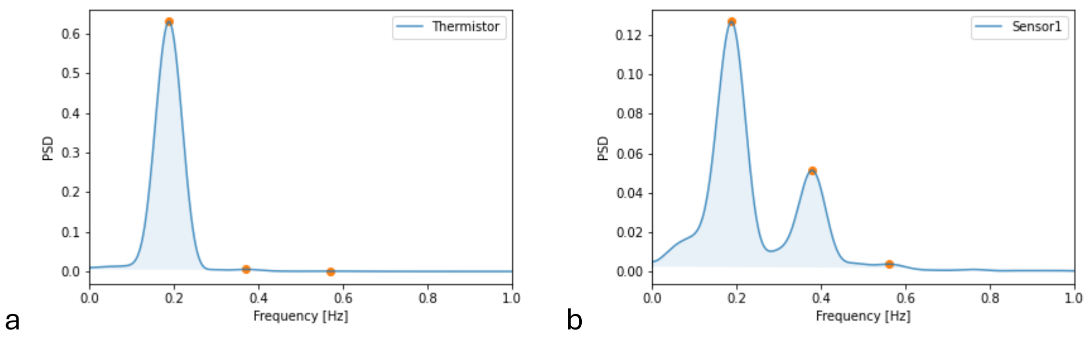

Figure S40: Demonstration of the PSD algorithm with the peaks and valleys that the algorithm found..(a) PSD for the 4 minute test of Thermistor for subject 3 (b) PSD for the 4 minute test of the Linear sensor with horizontal orientation on the left side of the ribs for subject 3.

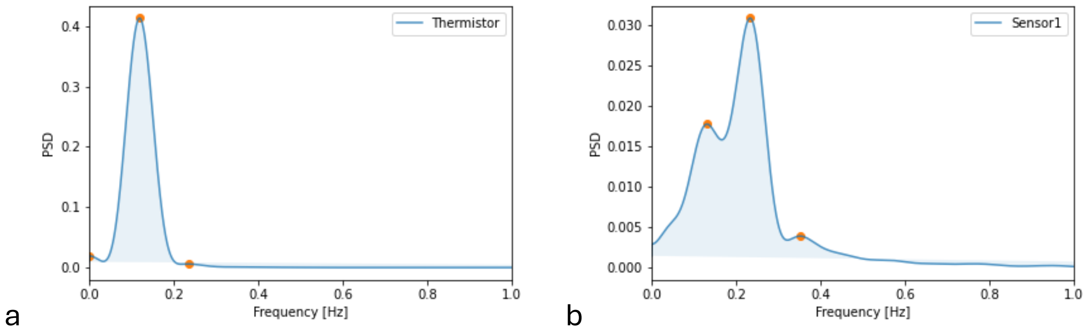

Figure S41: Demonstration of the PSD algorithm with the peaks and valleys that the algorithm found.(a) PSD for the 4 minute test of Thermistor for subject 1 (b) PSD for the 4 minute test of the Linear sensor with horizontal orientation on the center of the chest for subject 1.

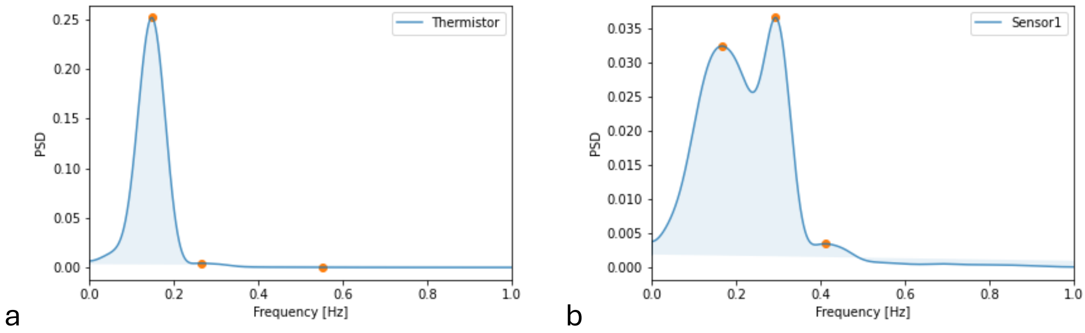

Figure S42: Demonstration of the PSD algorithm with the peaks and valleys that the algorithm found.(a) PSD for the 4 minute test of Thermistor for subject 2 (b) PSD for the 4 minute test of the Linear sensor with horizontal orientation on the center of the chest for subject 2.

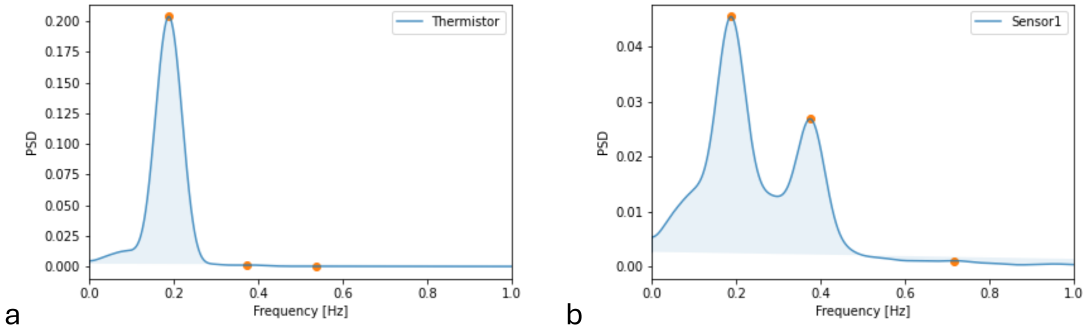

Figure S43: Demonstration of the PSD algorithm with the peaks and valleys that the algorithm found.(a) PSD for the 4 minute test of Thermistor for subject 3 (b) PSD for the 4 minute test of the Linear sensor with horizontal orientation on the center of the chest for subject 3.

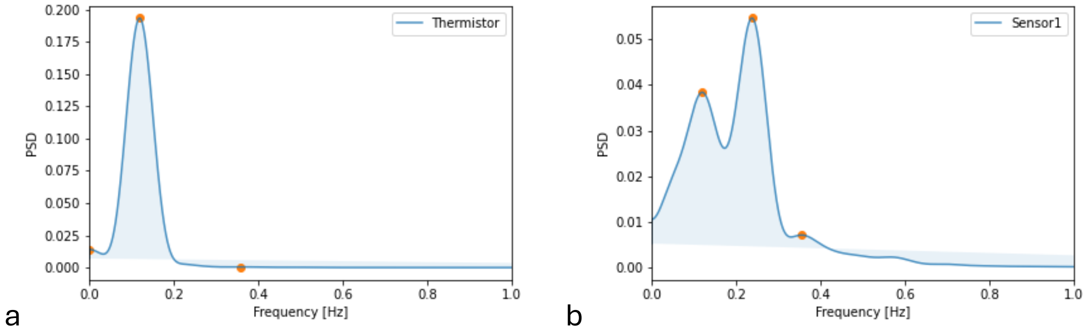

Figure S44: Demonstration of the PSD algorithm with the peaks and valleys that the algorithm found.(a) PSD for the 4 minute test of Thermistor for subject 1 (b) PSD for the 4 minute test of the Linear sensor with horizontal orientation on the right side of the ribs for subject 1.

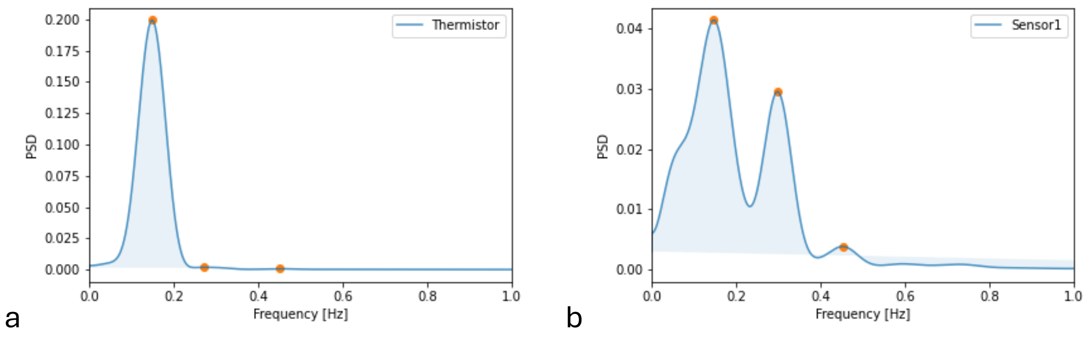

Figure S45: Demonstration of the PSD algorithm with the peaks and valleys that the algorithm found.(a) PSD for the 4 minute test of Thermistor for subject 2 (b) PSD for the 4 minute test of the Linear sensor with horizontal orientation on the right side of the ribs for subject 2.

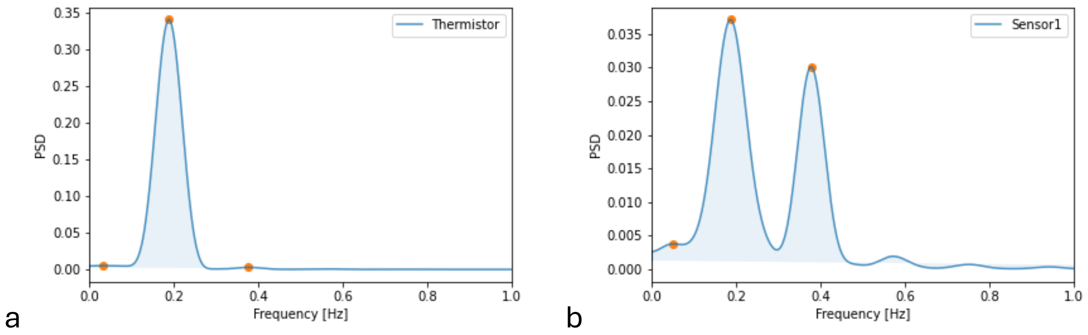

Figure S46: Demonstration of the PSD algorithm with the peaks and valleys that the algorithm found.(a) PSD for the 4 minute test of Thermistor for subject 3 (b) PSD for the 4 minute test of the Linear sensor with horizontal orientation on the right side of the ribs for subject 3.

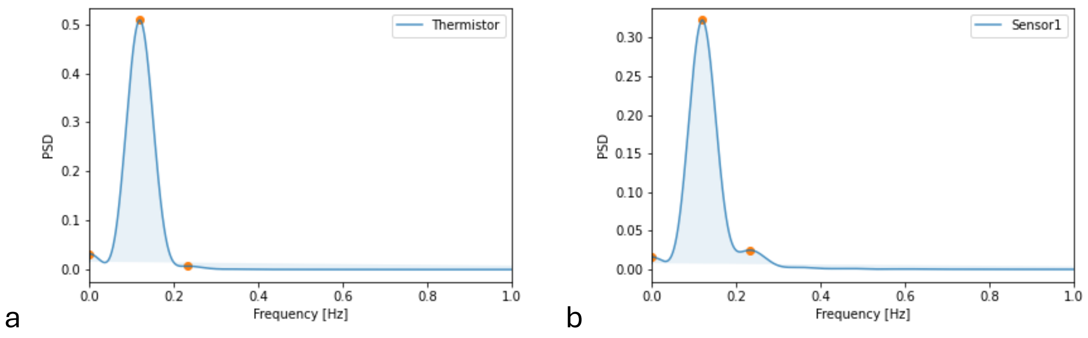

Figure S47: Demonstration of the PSD algorithm with the peaks and valleys that the algorithm found.(a) PSD for the 4 minute test of Thermistor for subject 2 (b) PSD for the 4 minute test of the Linear sensor with vertical orientation on the left side of the ribs for subject 2.

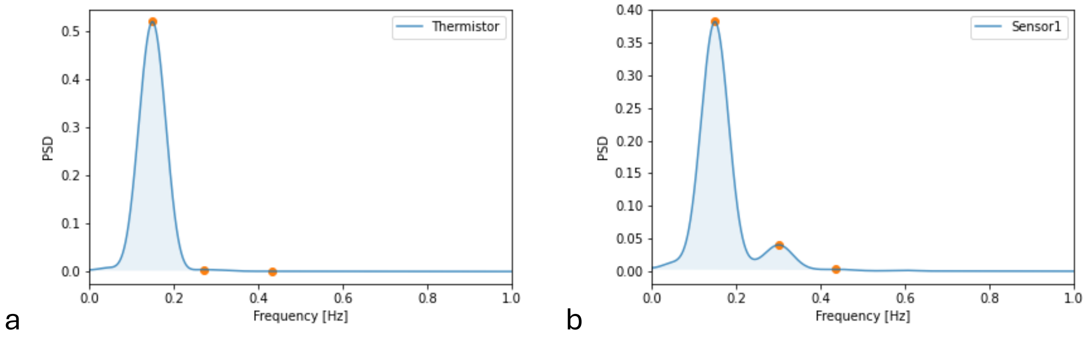

Figure S48: Demonstration of the PSD algorithm with the peaks and valleys that the algorithm found.(a) PSD for the 4 minute test of Thermistor for subject 2 (b) PSD for the 4 minute test of the Linear sensor with vertical orientation on the left side of the ribs for subject 2.

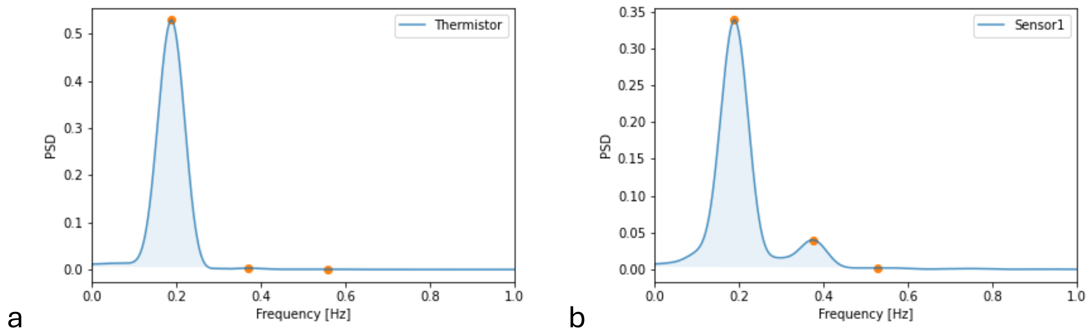

Figure S49: Demonstration of the PSD algorithm with the peaks and valleys that the algorithm found.(a) PSD for the 4 minute test of Thermistor for subject 3 (b) PSD for the 4 minute test of the Linear sensor with vertical orientation on the left side of the ribs for subject 3.

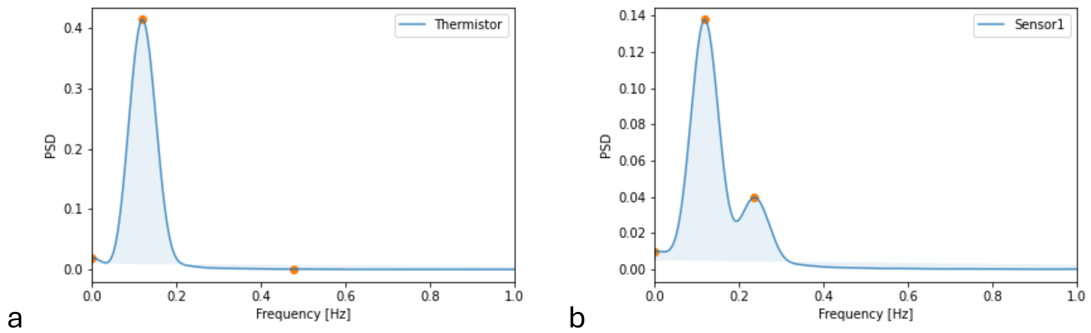

Figure S50: Demonstration of the PSD algorithm with the peaks and valleys that the algorithm found.(a) PSD for the 4 minute test of Thermistor for subject 1 (b) PSD for the 4 minute test of the Linear sensor with vertical orientation on the center of the chest for subject 1.

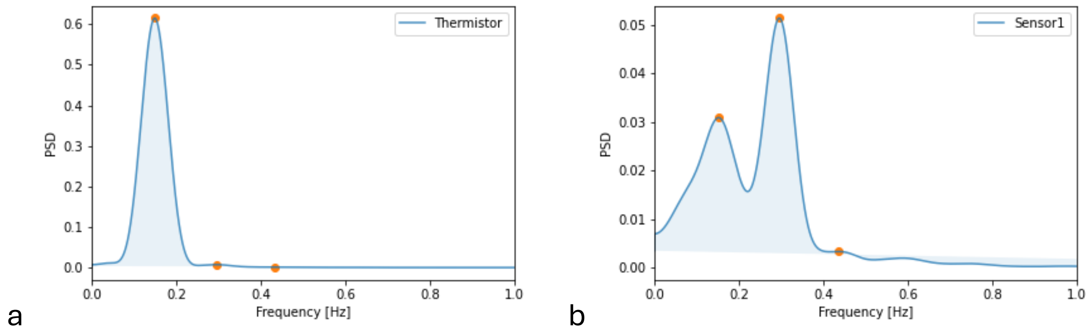

Figure S51: Demonstration of the PSD algorithm with the peaks and valleys that the algorithm found.(a) PSD for the 4 minute test of Thermistor for subject 2 (b) PSD for the 4 minute test of the Linear sensor with vertical orientation on the center of the chest for subject 2.

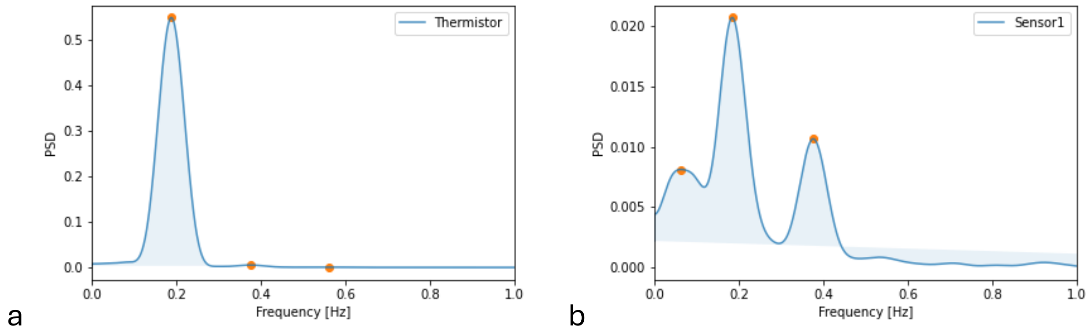

Figure S52: Demonstration of the PSD algorithm with the peaks and valleys that the algorithm found.(a) PSD for the 4 minute test of Thermistor for subject 3 (b) PSD for the 4 minute test of the Linear sensor with vertical orientation on the center of the chest for subject 3.

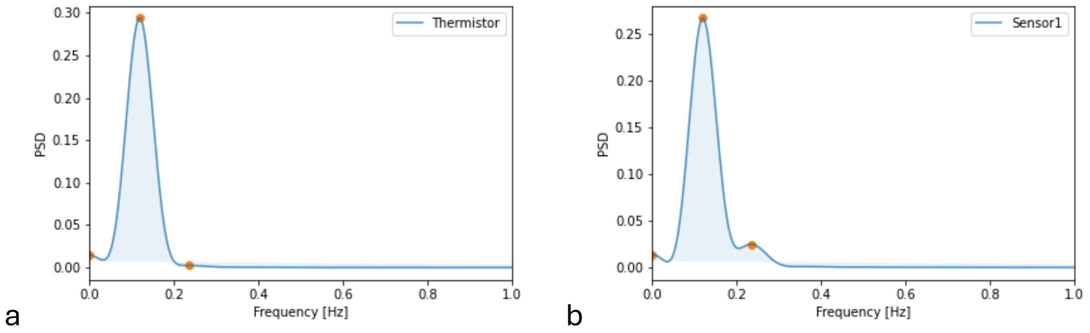

Figure S53: Demonstration of the PSD algorithm with the peaks and valleys that the algorithm found.(a) PSD for the 4 minute test of Thermistor for subject 1 (b) PSD for the 4 minute test of the Linear sensor with vertical orientation on the right side of the ribs for subject 1.

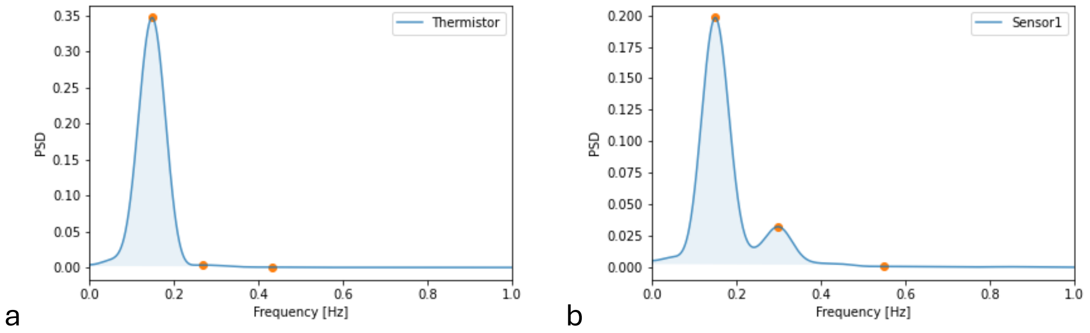

Figure S54: Demonstration of the PSD algorithm with the peaks and valleys that the algorithm found.(a) PSD for the 4 minute test of Thermistor for subject 2 (b) PSD for the 4 minute test of the Linear sensor with vertical orientation on the right side of the ribs for subject 2.

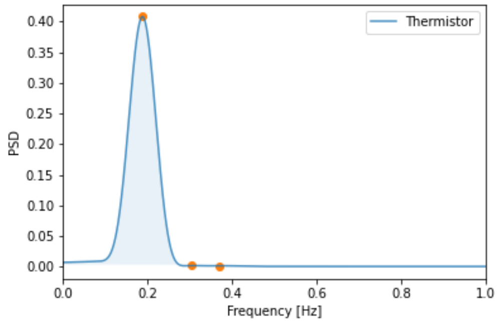

a

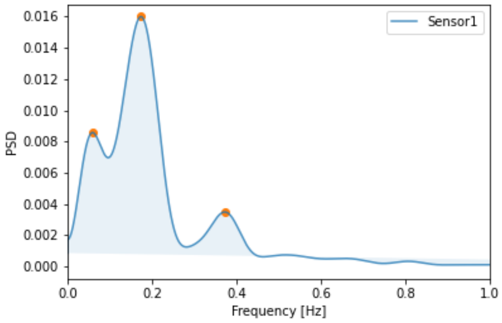

b

Figure S55: Demonstration of the PSD algorithm with the peaks and valleys that the algorithm found.(a) PSD for the 4 minute test of Thermistor for subject 3 (b) PSD for the 4 minute test of the Linear sensor with vertical orientation on the right side of the ribs for subject 3.

Table S4: Detailed view on the Circular sensor for the 4 minute test with the PSD algorythm.

Figure	Subject	Sensor Position	Thermistor Measure	Sensor Measure	Error
4.2	1	Circular on Left	7,40	10,44	3,04
B.1	2	Circular on Left	9,24	9,56	0,32
B.2	3	Circular on Left	10,30	9,12	1,18
B.3	1	Circular on Center	7,18	8,18	1,00
B.4	2	Circular on Center	9,05	8,35	0,70
B.5	3	Circular on Center	12,04	9,27	2,77
B.6	1	Circular on Right	8,04	10,30	2,26
B.7	2	Circular on Right	9,52	10,53	1,01
B.8	3	Circular on Right	11,33	9,39	

Table S5: Detailed view on the Linear sensor with the Horizontal Orientation for the 4 minute test with the PSD algorythm.

Figure	Subject	Sensor Position	Thermistor Measure	Sensor Measure	Error
B.9	1	Linear on Left	7,79	11,75	3,96
B.10	2	Linear on Left	9,13	10,18	1,05
B.11	3	Linear on Left	11,28	11,92	0,64
B.12	1	Linear on Center	7,31	10,08	2,77
B.13	2	Linear on Center	9,09	10,81	1,72
B.14	3	Linear on Center	11,33	10,39	0,94
B.15	1	Linear on Right	8,00	13,16	5,16
B.16	2	Linear on Right	9,28	9,94	0,66
B.17	3	Linear on Right	11,42	10,50	0,92

Table S6: Detailed view on the Linear sensor with the Vertical Orientation for the 4 minute test with the PSD algorythm.

**Figure**	**Subject**	**Sensor Position**	**Thermistor Measure**	**Sensor Measure**	**Error**
B.18	1	Linear on Left	7,73	7,18	0,55
B.19	2	Linear on Left	9,17	9,09	0,08
B.20	3	Linear on Left	12,57	11,89	0,68
B.21	1	Linear on Center	8,38	9,18	0,80
B.22	2	Linear on Center	9,05	9,44	0,39
B.23	3	Linear on Center	11,28	8,20	3,08
B.24	1	Linear on Right	7,96	8,18	0,22
B.25	2	Linear on Right	9,05	10,40	1,35
B.26	3	Linear on Right	12,09	7,24	4,85
